# Population and evolutionary dynamics in spatially structured seasonally varying environments

**DOI:** 10.1111/brv.12409

**Published:** 2018-03-25

**Authors:** Jane M. Reid, Justin M. J. Travis, Francis Daunt, Sarah J. Burthe, Sarah Wanless, Calvin Dytham

**Affiliations:** ^1^ School of Biological Sciences University of Aberdeen, Zoology Building, Tillydrone Avenue Aberdeen AB24 2TZ U.K.; ^2^ Centre for Ecology & Hydrology, Bush Estate, Penicuik Midlothian EH26 0QB U.K.; ^3^ Department of Biology University of York, Heslington York YO10 5DD U.K.

**Keywords:** demographic structure, density‐dependence, eco‐evolutionary dynamics, life‐history variation, meta‐population, movement ecology, partial migration, plasticity, population viability, seasonal migrant, vital rate

## Abstract

Increasingly imperative objectives in ecology are to understand and forecast population dynamic and evolutionary responses to seasonal environmental variation and change. Such population and evolutionary dynamics result from immediate and lagged responses of all key life‐history traits, and resulting demographic rates that affect population growth rate, to seasonal environmental conditions and population density. However, existing population dynamic and eco‐evolutionary theory and models have not yet fully encompassed within‐individual and among‐individual variation, covariation, structure and heterogeneity, and ongoing evolution, in a critical life‐history trait that allows individuals to respond to seasonal environmental conditions: seasonal migration. Meanwhile, empirical studies aided by new animal‐tracking technologies are increasingly demonstrating substantial within‐population variation in the occurrence and form of migration *versus* year‐round residence, generating diverse forms of ‘partial migration’ spanning diverse species, habitats and spatial scales. Such partially migratory systems form a continuum between the extreme scenarios of full migration and full year‐round residence, and are commonplace in nature.

Here, we first review basic scenarios of partial migration and associated models designed to identify conditions that facilitate the maintenance of migratory polymorphism. We highlight that such models have been fundamental to the development of partial migration theory, but are spatially and demographically simplistic compared to the rich bodies of population dynamic theory and models that consider spatially structured populations with dispersal but no migration, or consider populations experiencing strong seasonality and full obligate migration. Second, to provide an overarching conceptual framework for spatio‐temporal population dynamics, we define a ‘partially migratory meta‐population’ system as a spatially structured set of locations that can be occupied by different sets of resident and migrant individuals in different seasons, and where locations that can support reproduction can also be linked by dispersal. We outline key forms of within‐individual and among‐individual variation and structure in migration that could arise within such systems and interact with variation in individual survival, reproduction and dispersal to create complex population dynamics and evolutionary responses across locations, seasons, years and generations. Third, we review approaches by which population dynamic and eco‐evolutionary models could be developed to test hypotheses regarding the dynamics and persistence of partially migratory meta‐populations given diverse forms of seasonal environmental variation and change, and to forecast system‐specific dynamics. To demonstrate one such approach, we use an evolutionary individual‐based model to illustrate that multiple forms of partial migration can readily co‐exist in a simple spatially structured landscape. Finally, we summarise recent empirical studies that demonstrate key components of demographic structure in partial migration, and demonstrate diverse associations with reproduction and survival. We thereby identify key theoretical and empirical knowledge gaps that remain, and consider multiple complementary approaches by which these gaps can be filled in order to elucidate population dynamic and eco‐evolutionary responses to spatio‐temporal seasonal environmental variation and change.

## I. INTRODUCTION

Almost all wild populations utilise geographical ranges where environmental capabilities to support key life‐history stages, and hence to maintain demographic rates that underlie population growth rate, vary both spatially and temporally. The ubiquitous spatial variation in habitat and environmental suitability is overlain by stochastic among‐year variation within some typical range, plus occasional atypical extreme environmental events that can substantially impact key demographic rates (Thomas & Kunin, 1999; Jentsch, Kreyling, & Beierkuhnlein, 2007; Van de Pol *et al.,* 2010; Lawson *et al.,* 2015; Selwood, Mcgeoch, & Macnally, 2015; Bailey & Van de Pol, 2016; Sæther *et al.,* 2016). Most populations also experience some degree of predictable within‐year variation in environmental conditions stemming from seasonality, creating cyclic temporal variation in the capacity of different locations to support different life‐history activities (Caswell, 2001; Faaborg *et al.,* 2010; Morrison & Bolger, 2012; Small‐Lorenz *et al.,* 2013; Dingle, 2014). Spatial and within‐year temporal dynamics of environmental conditions, and associated demography, are then intrinsically linked. Critical objectives in ecology are consequently to identify general principles that underlie the short‐term and longer‐term spatio‐temporal dynamics of populations utilising spatially structured seasonally varying environments, and hence to understand and forecast population responses to spatio‐temporal seasonal environmental change (Runge & Marra, 2005; Fryxell & Holt, 2013; Small‐Lorenz *et al.,* 2013; Selwood *et al.,* 2015; Rushing *et al.,* 2017; Van de Pol *et al.,* 2017). These objectives are increasingly imperative because climate models predict widespread changes in means and variances in seasonal conditions and hence in the degree of seasonality, and predict increasing frequencies, magnitudes and durations of extreme seasonal climatic events (e.g. storms, heatwaves, intense rainfall; Easterling *et al.,* 2000; Ummenhofer & Meehl, 2017). Such changes could substantially impact location‐specific and season‐specific demography, and thereby ameliorate or exacerbate current seasonal constraints on population demography, dynamics, range and persistence (e.g. Jentsch *et al.,* 2007; Welbergen *et al.,* 2008; Van de Pol *et al.,* 2010; Selwood *et al.,* 2015; Bailey & Van de Pol, 2016).

Against this backdrop, some overarching principles of population‐dynamic responses to environmental variation are well established. In general, spatio‐temporal population dynamics depend on immediate and lagged (delayed) effects of typical ranges of environmental variation, and of atypical extreme events, on all key demographic rates that affect population growth rate (i.e. ‘vital rates’; Ådahl, Lundberg, & Jonzén, 2006; Benton, Plaistow, & Coulson, 2006; Van de Pol *et al.,* 2010; Lawson *et al.,* 2015; Selwood *et al.,* 2015; Gamelon *et al.,* 2017). Such effects result from individuals' life‐history responses to environmental conditions and population density (i.e. density‐dependence), including the forms and magnitudes of carry‐over effects and developmental and environmental canalisation *versus* plasticity (Pfister, 1998; Beckerman *et al.,* 2002; Ratikainen *et al.,* 2008; Harrison *et al.,* 2011; Sæther *et al.,* 2016). Further, key life‐history traits are rarely uniformly expressed by all population members and rarely vary independently, generating distinct means, variances and covariances within and across different subsets of individuals structured by sex, age, stage, state and/or cohort (e.g. Van Tienderen, 2000; Caswell, 2001; Lindström & Kokko, 2002; Benton *et al.,* 2006; Sæther *et al.,* 2013; Lawson *et al.,* 2015). Resulting complex forms of life‐history and demographic variation, covariation, structure and heterogeneity have been shown to substantively affect population dynamics (Beckerman *et al.,* 2002; Clutton‐Brock & Coulson, 2002; Lindström & Kokko, 2002; Coulson, Gaillard & Festa‐Bianchet, 2005; Doak *et al.,* 2005; Benton *et al.,* 2006; Vindenes, Engen, & Sæther, 2008; Sæther *et al.,* 2013; Lawson *et al.,* 2015).

In addition, long‐term population dynamics manifested across multiple generations will also depend on the degrees to which key life‐history trait means, variances, covariances and plasticities evolve in response to changing means, variances and extremes in environmental conditions (Benton *et al.,* 2006; Bailey & Van de Pol, 2016; Chevin & Hoffmann, 2017). Such evolution might be rapid, and hence non‐trivial on ecological timescales (Pelletier, Garant, & Hendry, 2009; Bonte *et al.,* 2012; Gonzalez *et al.,* 2013; Legrand *et al.,* 2017). Overall, therefore, theoretical and empirical studies aiming to understand observed spatio‐temporal population dynamics, and forecast future dynamics, must encompass sufficient complexity in the forms of current and evolving life‐history variation and covariation in relation to spatio‐temporal seasonal variation in environmental conditions and population density (Clutton‐Brock & Coulson, 2002; Runge & Marra, 2005; Benton *et al.,* 2006; Ratikainen *et al.,* 2008; Van de Pol *et al.,* 2010; Travis *et al.,* 2012; Sæther *et al.,* 2013; Lawson *et al.,* 2015; Gamelon *et al.,* 2017).

However, despite such well‐established overarching ambitions and principles, major bodies of population dynamic theory, and empirical studies, have not yet fully encompassed major components of within‐individual and among‐individual variation, covariation, structure, heterogeneity and evolution in a critical life‐history trait that allows individuals to respond to spatio‐temporal seasonal environmental change: seasonal migration.

### (1) Spatio‐temporal population dynamics in seasonally varying environments

In general, spatio‐temporal population dynamics can be quantified as time series of the number and composition of individuals inhabiting each focal location across sequences of consecutive seasons, or in the same season across consecutive years. Given seasonal variation in environmental conditions and population density and resulting demography, among‐year dynamics will ultimately depend on among‐season (i.e. within‐year) dynamics (Sutherland & Dolman, 1994; Caswell, 2001; Runge & Marra, 2005; Ratikainen *et al.,* 2008; Holt & Fryxell, 2011; Hostetler, Sillett & Marra, 2015; Rushing *et al.,* 2017). Such dynamics will in turn depend on immediate and lagged variation and covariation in four key life‐history traits expressed by individuals and resulting demographic rates emerging across sets of individuals: reproduction, survival, dispersal and seasonal migration. Here, dispersal is defined as movements of individuals among natal and subsequent breeding locations between breeding seasons (and hence often between years), resulting in local emigration and immigration between reproductive events (Webster *et al.,* 2002; Bonte *et al.,* 2012; Cote *et al.,* 2017; Legrand *et al.,* 2017). Meanwhile, seasonal migration is, for current purposes, most simply defined as reversible movements of individuals between distinct breeding and non‐breeding locations and seasons, meaning that reproduction does not typically occur between outward and return migrations (Webster *et al.,* 2002; Newton, 2008; Faaborg *et al.,* 2010; Shaw & Couzin, 2013; Peters *et al.,* 2017, but see Dingle & Drake, 2007; Morita *et al.,* 2014; Cote *et al.,* 2017; Harrison *et al.,* 2017 and Section V.2).

Accordingly, annual reproduction, survival and dispersal together describe the local productivity and persistence of individuals within locations across years and are consequently the primary determinants of among‐year spatio‐temporal population dynamics (Thomas & Kunin, 1999; Neubert & Caswell, 2000; Selwood *et al.,* 2015; Gamelon *et al.,* 2017). However, seasonal migration can also play major roles, both as a primary demographic rate and as a mechanistic structuring process that can affect reproduction, survival and dispersal. These roles stem from the fact that migration is a critical life‐history trait that evolves to allow individuals to anticipate or respond to spatio‐temporal seasonal environmental variation. Migration allows individuals to increase their survival and/or reproduction by exploiting spatially restricted seasonal peaks in resource availability while avoiding seasonally inhospitable local environments or mitigating disease or predation risk, and can thereby increase overall population size and density (e.g. Pulido, 2007; Faaborg *et al.,* 2010; Griswold, Taylor, & Norris, 2011; Skov *et al.,* 2013; Avgar, Street, & Fryxell, 2014; Dingle, 2014; Liedvogel & Lundberg, 2014; Eggeman *et al.,* 2016; Shaw & Binning, 2016).

First, by definition, migration redistributes individuals in space among seasons and consequently directly and profoundly affects among‐season (i.e. commonly within‐year) spatio‐temporal population dynamics. Second, because migration can require physiological transitions and affects individuals' environmental experiences and location‐specific population densities, it can directly affect survival and create carry‐over effects that influence subsequent reproduction and exacerbate pre‐existing heterogeneities in individual life histories (e.g. Gunnarsson *et al.,* 2005; Harrison *et al.,* 2011). It can thereby substantively affect among‐year population dynamics (e.g. Runge & Marra, 2005; Norris & Taylor, 2006; Ratikainen *et al.,* 2008; Faaborg *et al.,* 2010). Third, the occurrence or form of migration can vary within and among individuals and rapidly evolve in response to covariances with survival and reproduction (i.e. selection), potentially creating complex evolutionary dynamics on ecological timescales (Pulido *et al.,* 2001; Van Noordwijk *et al.,* 2006; Pulido, 2007; Pulido & Berthold, 2010). Overall, therefore, migration constitutes a major, flexible (i.e. plastic) and evolving life‐history trait and structuring process that could have multiple immediate, lagged and long‐term effects on the demography and dynamics of populations inhabiting seasonally varying environments (Sutherland & Dolman, 1994; Gunnarsson *et al.,* 2005; Runge & Marra, 2005; Norris & Taylor, 2006; Hostetler *et al.,* 2015).

The need to explicitly incorporate seasonality and associated seasonal migration and demography into population dynamic theory and forecasts has been emphasised as an urgent goal in the face of projected seasonal environmental changes (Sutherland & Dolman, 1994; Webster *et al.,* 2002; Runge & Marra, 2005; Norris & Taylor, 2006; Faaborg *et al.,* 2010; Small‐Lorenz *et al.,* 2013; Marra *et al.,* 2015). However, despite recent advances, we still lack comprehensive population dynamic models and empirical studies that consider the full spectrum of within‐individual and among‐individual variation, covariation, structure and heterogeneity in migration alongside survival, reproduction and dispersal.

### (2) Population dynamic theory and models that do not consider seasonal migration

Long‐standing bodies of general theory that consider population and evolutionary dynamics stemming from complex forms of life‐history and demographic variation and structure often do not explicitly consider seasonality, or associated seasonal migration, at all. Rather, models that consider effects of environmental variation and population density on demographic rates and structures, and hence on deterministic or stochastic population growth rates, initially focused on reproduction and survival (and sometimes on underlying growth, development or phenology) as the sole demographic processes (e.g. Grant & Benton, 2000; Van Tienderen, 2000; Caswell, 2001; Lindström & Kokko, 2002; Hodgson & Townley, 2004; Ådahl *et al.,* 2006; Ezard *et al.,* 2010; Sæther *et al.,* 2013, 2016; Lawson *et al.,* 2015; McDonald *et al.,* 2016; Salguero‐Gómez *et al.,* 2016). Such work quantifies effects of small perturbations in demographic rates (e.g. sensitivities, elasticities), and larger perturbations and resulting transient dynamics (e.g. reactivities), given different life histories spanning the fecundity–survival spectrum. Results apply directly to single resident populations, and can be applied indirectly to spatially structured or seasonally mobile populations if effects of dispersal and migration are implicitly subsumed into variation in local annual survival and reproduction (and underlying density). However, such theory and models cannot explicitly consider spatial population dynamics, or hence directly forecast range dynamics or identify key seasonal locations that underpin overall population dynamics and persistence.

Consequently, further substantial bodies of work have explicitly considered population dynamics in patchy or spatially heterogeneous habitats, stemming from variation in dispersal alongside (implicit or explicit) variation in reproduction and survival. For example, classical meta‐population theory and stochastic patch occupancy models quantify the consequences of patch sizes and separations and associated dispersal rates for probabilities of patch‐population extinction and recolonisation and resulting meta‐population dynamics and persistence; this approach transformed the conceptualisation of spatio‐temporal population dynamics (e.g. Hanski, 1999; Sutherland, Elston, & Lambin, 2014). Matrix models can explicitly consider dispersal rates among patches that support different rates of reproduction and survival, and thereby evaluate scenarios of local habitat destruction or creation (e.g. Caswell, Lensink, & Neubert, 2003; Strasser *et al.,* 2012). Joint matrix and integro‐difference equation models allow sensitivity analyses pertaining to population invasion speeds and range shifts (Neubert & Caswell, 2000; Bullock *et al.,* 2012), including in heterogeneous landscapes (Gilbert *et al.,* 2014). Meanwhile, spatially explicit individual‐based models (IBMs) can include mechanistic representations of dispersal, encompassing context‐, sex‐ and stage‐dependent individual decisions and costs concerning the sequential phases of departure, transfer and settlement (Travis *et al.,* 2012; Bocedi *et al.,* 2014; Aben *et al.,* 2016). Dispersal rates, distances and directions, and resulting spatial population dynamics, then emerge from underlying ecologically informed individual decisions rather than being constrained to imposed values or distributions (Bocedi *et al.,* 2014). Such IBMs can also readily track evolutionary dynamics and postulated drivers of dispersal, including kin competition, inbreeding and bet‐hedging, and thereby test eco‐evolutionary hypotheses (Travis *et al.,* 2012).

Such matrix models and IBMs that link structured variation in survival, reproduction and dispersal to spatial population dynamics have greatly facilitated general theory development and system‐specific forecasting of population viability and range expansion in relation to spatially explicit scenarios of environmental change (e.g. Bullock *et al.,* 2012; Lurgi *et al.,* 2015; Aben *et al.,* 2016; Legrand *et al.,* 2017). However, leading general modelling frameworks that explicitly consider multi‐patch or complex landscapes with structured or context‐dependent dispersal, and associated individual variation in survival and reproduction, have not yet also considered variation and structure in seasonal migration or associated seasonal demography and dynamics. Such models consequently still ignore a major dimension of life‐history variation that arises within and among individuals, and resulting demographic variation, covariation, structure and heterogeneity, that could substantially shape short‐term and longer‐term population dynamic responses to spatio‐temporal seasonal environmental change.

### (3) Population dynamic models that consider obligate seasonal migration

The recognition that models that ignore seasonal environmental variation and demography might forecast erroneous population dynamics, or incorrectly identify key locations for population persistence, has prompted repeated calls to build, parameterise and analyse ‘full annual cycle’ models that explicitly consider seasonality (Sutherland & Dolman, 1994; Webster *et al.,* 2002; Runge & Marra, 2005; Norris & Taylor, 2006; Small‐Lorenz *et al.,* 2013; Hostetler *et al.,* 2015; Marra *et al.,* 2015). Consequently, diverse mathematical, meta‐population, matrix, network and individual‐based models have been constructed that explicitly include seasonal migration as a structural process that links demography across seasonal environments, with internally consistent seasonal density‐dependence and carry‐over effects on reproduction and/or survival (e.g. Sutherland & Dolman, 1994; Runge & Marra, 2005; Norris & Taylor, 2006; Taylor & Norris, 2010; Taylor & Hall, 2012; Gilroy *et al.,* 2016; reviewed by Hostetler *et al.,* 2015).

However, such models and associated analyses have not yet fully considered sex‐, age‐, stage‐, state‐, cohort‐ and/or location effects on the form or occurrence of migration, and hence on other associated demographic rates (Runge & Marra, 2005; Small‐Lorenz *et al.,* 2013; Hostetler *et al.,* 2015). Indeed, most ‘full annual cycle’ models designed to explore population dynamics treat migration as an obligate transition and fixed structural process: all individuals migrate (or die). They thereby typically assume complete strong seasonality such that breeding locations cannot support non‐breeding‐season survival and non‐breeding‐season locations cannot support reproduction, fostering obligate directional migration (Webster *et al.,* 2002; Taylor & Norris, 2010; Hostetler *et al.,* 2015). Consequently, such models have not yet fully considered population dynamics stemming from spatial, temporal and individual variation in the occurrence of migration *versus* residence (i.e. partial migration), and associated covariances with reproduction, survival or dispersal. Further, such models have not generally considered plasticity or short‐term evolutionary dynamics of migration *versus* residence, or resulting eco‐evolutionary feedbacks that could fundamentally affect population dynamic responses to environmental change.

In an evolutionary context, Shaw & Couzin (2013) used a spatially explicit IBM to identify forms of information use and selection under which directional migration (as opposed to residence) evolved in complex patchy landscapes. Migration typically evolved when habitats were more seasonal than patchy, to degrees that also depended on the forms of available information and the fitness benefits of migration (Shaw & Couzin, 2013). Guttall & Couzin (2010) also used a spatially explicit IBM to consider evolution of migration as a collective, socially informed behaviour (reviewed by Cote *et al.,* 2017). Here, migration evolved readily in the presence of a constant global gradient that could be detected with little error, generating co‐existing ‘leaders’ and ‘followers’ that migrated using direct and social information, respectively. However, Shaw & Couzin (2013) considered outward migratory movements only, and Guttall & Couzin (2010) did not explicitly consider spatial habitat heterogeneity. Neither model considered any form of demographic structure in relation to seasonal environmental variation and associated fitness costs or benefits of migration, or explicitly examined any emerging partial migration or population dynamics or persistence (Guttall & Couzin, 2010).

Overall, therefore, population dynamic models for (potentially) migratory populations have not yet fully embraced major forms of demographic structure and variation that are known to substantially affect the dynamics of non‐migratory populations, and hence are unlikely to be ignorable. Nor have they embraced key forms of context‐dependent (i.e. plastic) and evolving individual variation in migration (and underlying departure, transfer and settlement) analogous to those that are increasingly central to mechanistic modelling and forecasting for spatial population dynamics involving dispersal (e.g. Bocedi *et al.,* 2014; Lurgi *et al.,* 2015; Cote *et al.,* 2017; Legrand *et al.,* 2017).

### (4) Objectives

Progress in understanding and forecasting the dynamics of populations inhabiting spatially structured seasonally varying environments now requires new models and empirical studies that coalesce attributes of the major existing bodies of work that consider complex structure, variation and micro‐evolution in key demographic rates in non‐migratory systems (Section I.2), or consider seasonal demography given obligate seasonal migration (Section I.3). By fully encompassing demographic complexity involving seasonality and among‐individual and within‐individual variation in seasonal migration, such work could provide a holistic framework for population dynamic theory and forecasting.

To facilitate this goal, we first review fundamental scenarios where the occurrence and form of seasonal migration varies among individuals within populations, creating different forms of spatio‐temporal population structure (Sections II and III). Second, to provide an overarching general framework that encompasses all these scenarios, we outline the concept of a ‘partially migratory meta‐population’ (PMMP). We draw on this framework to hypothesise numerous ways in which complex forms of spatio‐temporal structure and variation in migration within and among individuals could arise and interact with variation in reproduction, survival and dispersal to shape population dynamics across locations, seasons, years and generations (Section IV). Finally, we provide agendas for new population dynamic and demographic theory, models and empirical studies that are required to address emerging hypotheses and questions and, ultimately, to fulfil the urgent requirement to understand and forecast population dynamics in spatially structured seasonally varying environments (Sections V and VI).

## II. SEASONAL MIGRATION AS A VARIABLE LIFE‐HISTORY TRAIT AND DEMOGRAPHIC RATE

Some populations are well known to be fully (i.e. obligately) seasonally migratory, where all individuals undertake directional outward and return movements between distinct geographical locations between distinct breeding and non‐breeding seasons (Newton, 2008; Faaborg *et al.,* 2010; Dingle, 2014). Within such systems, different individuals can migrate between different initial and destination locations in ‘leapfrog’, ‘chain’ or ‘telescopic’ structures with different degrees of migratory dispersion and connectivity (Webster *et al.,* 2002; Taylor & Norris, 2010; Gilroy *et al.,* 2016). The form of migration, and the life‐history and demographic consequences, can consequently vary substantially among individuals and sub‐populations that experience different seasonal environmental conditions and transitions (e.g. Norris *et al.,* 2004; Gunnarsson *et al.,* 2005; Runge & Marra, 2005; Flack *et al.,* 2016; Lok *et al.,* 2017).

**Figure 1:**
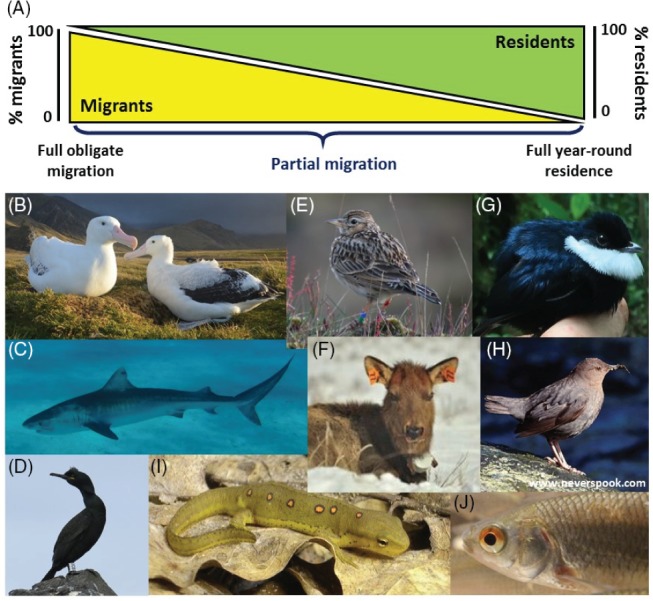
(A) Illustration that seasonal partial migration forms a continuum between the extreme scenarios of full obligate migration and full year‐round residence. (B–J) Examples of diverse partially migratory taxa. Migratory individuals can make long‐ or medium‐distance geographical migrations (B–E), or medium‐distance altitudinal migrations (F–H), or short‐distance migrations between adjacent but distinct habitat types (I, J). Geographical migrations occur in (B) wandering albatross (*Diomedea exulans*; Weimerskirch *et al.,* 2015); (C) tiger shark (*Galeocerdo cuvier*; Papastamatiou *et al.,* 2013); (D) European shag (*Phalacrocorax aristotelis*; Grist *et al.,* 2014, 2017); (E) Skylark (*Alauda arvensis*; Hegemann *et al.,* 2015), and numerous other birds [including blackbirds, *Turdus merula* (Fudickar *et al.,* 2013; Zúñiga *et al.,* 2017), and American kestrels, *Falco sparverius* (Anderson *et al.,* 2015)]. Altitudinal migrations occur in (F) elk (*Cervus elaphus*; Hebblewhite & Merrill, 2011; Eggeman *et al.,* 2016) and other ungulates (e.g. *caribou Rangifer tarandus*; McDevitt *et al.,* 2009), and in (G) white‐ruffed manakin (*Corapipo altera*; Boyle *et al.,* 2010, 2011) and (H) American dipper (*Cinclus mexicanus*; Gillis *et al.,* 2008; Green *et al.,* 2015) and numerous other birds (Boyle, 2017). Habitat‐related migrations occur in (I) red‐spotted newt (*Notophthalmus viridescens*, Grayson & Wilbur, 2009; Grayson *et al.,* 2011) and (J) roach (*Rutilus rutilus*; Brodersen *et al.,* 2008; Skov *et al.,* 2013) and many other fish (e.g. Chapman *et al.,* 2012; Vélez‐Espino *et al.,* 2013), and also ungulates such as roe deer (*Capreolus capreolus*; Peters *et al.,* 2017). Seasonal partial migration across diverse spatial scales also occurs in reptiles (e.g. Shaw & Levin, 2011; Yackulic *et al.,* 2017). Partial migration can also occur on shorter timeframes, including diel migrations observed in fish and invertebrates (e.g. Chapman *et al.,* 2011; Harrison *et al.,* 2017). Photograph credits: (B) Henri Weimerskirch; (C) Yannis Papastamatiou; (D) Mark Newell; (E) Rob Voesten; (F) Celie Intering; (G) Alice Boyle; (H) Roberta Olenick; (I) Kristine Grayson; (J) Jakob Brodersen.

However, it is increasingly evident that ‘partial migration’, where single populations contain mixtures of seasonally migrant individuals and year‐round residents, occurs very widely and may even predominate in nature (Fig. 1; Lundberg, 1988; Chapman *et al.,* 2011). Facilitated by technological advances that allow individual animals to be tracked across seasons, diverse forms of seasonal partial migration have been documented in numerous fish, birds, mammals, amphibians and reptiles, in temperate and tropical regions, and spanning terrestrial, freshwater and marine environments (e.g. Fig. 1; Berthold, 1999; Newton, 2008; Chapman *et al.,* 2011, 2012; Shaw & Levin, 2011; Avgar *et al.,* 2014; Dingle, 2014; Boyle, 2017; Peters *et al.,* 2017). Such partial migration encompasses cases where migratory individuals undertake long‐distance geographical migrations to single or multiple destinations, while other individuals remain resident. It also encompasses cases where migratory individuals undertake medium‐ or short‐distance seasonal migrations across altitudinal gradients or between adjacent habitat types, meaning that migration is not necessarily uniformly geographically directional. Partial migration therefore spans hugely diverse species, ecologies, life histories and spatial scales, and spans the continuum between the extreme scenarios of full obligate migration and full residence, both of which may in fact be relatively unusual (Fig. 1; Berthold, 1999; Dingle & Drake, 2007; Pulido, 2007; Chapman *et al.,* 2011, 2012; Shaw & Levin, 2011; Boyle, 2017; Peters *et al.,* 2017).

Partial migration can be viewed as the population‐level outcome of an underlying axis of individual variation that translates into a qualitative state difference between residence and migration that each individual expresses at any point in time (Pulido, 2007, 2011). Such partial migration can act alongside differences among migratory individuals that move to different destinations to generate population‐wide demographic structure and variation (e.g. Gurarie *et al.,* 2017; Peters *et al.,* 2017). By definition, partial migration means that current residents remain in single locations while current migrants experience different physiological and ecological processes linked to movement and the fact that they inhabit different locations across seasons. Migrants *versus* residents might consequently experience substantial differences and discontinuities in physiology, in environmental conditions and information, in territory occupancy, social interactions and competition, and in energy, predation risk or parasitism costs or benefits stemming directly from departure and/or subsequent movement and/or settlement into new locations (e.g. Kokko & Lundberg, 2001; Olsson *et al.,* 2006; Grayson & Wilbur, 2009; Griswold *et al.,* 2011; Kokko, 2011; Avgar *et al.,* 2014; Shaw & Binning, 2016; Yackulic, Blake & Bastille‐Rousseau, 2017). These differences mirror those experienced by dispersers *versus* non‐dispersers (Bonte *et al.,* 2012; Travis *et al.,* 2012; Cote *et al.,* 2017), and could substantially affect current or future reproduction, survival and/or dispersal. Partial migration could thereby create degrees of life history and demographic variation, covariation, structure and heterogeneity that exceed those arising in fully resident populations, or in fully migratory populations where all individuals experience relatively similar seasonal discontinuities in physiology, environment, information and territoriality and direct costs or benefits of departure, even if migrants move to diverse locations (Lundberg, 1988).

Further, expression of migration *versus* residence can vary with individual state and local environmental conditions, generating phenotypic plasticity such that individuals switch between residence and migration at different points in time (e.g. Brodersen *et al.,* 2008; Grayson & Wilbur, 2009; Fudickar *et al.,* 2013; Eggeman *et al.,* 2016; Peters *et al.,* 2017). Such plasticity can potentially generate density‐dependence in the occurrence or form of migration (Brodersen *et al.,* 2008; Grayson & Wilbur, 2009; Eggeman *et al.,* 2016). It can also generate ‘irruptive’ migration that occurs as a facultative response to extreme seasonal environmental conditions rather than as a pre‐emptive (anticipatory) action preceding predictable seasonal environmental change (Newton, 2008; Boyle, Norris, & Guglielmo, 2010; Lindén *et al.,* 2011). Conversely, an individual's strategy of migration or residence could be strongly genetically determined or developmentally or environmentally canalised, and hence consistently and inflexibly expressed across different environmental conditions experienced in different years. The degree of plasticity or canalisation could in turn have a genetic basis. Both baseline migration propensity and the form of plasticity or canalisation could then evolve in response to selection on the expression of migration stemming from spatio‐temporal environmental change. Such evolution could be rapid given substantial additive genetic variation, and given strong selection stemming from extreme environmental events that cause high mortality or prevent reproduction in some seasons and locations and thereby impact specific spatially segregated sets of migrants or residents (e.g. Berthold, 1999; Pulido *et al.,* 2001; Pulido & Berthold, 2010; Liedvogel, Åkesson, & Bensch, 2011; Páez *et al.,* 2011). Such dynamic, heterogeneous and evolving partial migration could profoundly affect population structure, demography and dynamics, including short‐term and longer‐term eco‐evolutionary responses to changing means, variances and extremes in seasonal environmental conditions.

However, despite the ubiquity of complex spatial variation in seasonal environmental conditions, increasingly widespread evidence of diverse forms of partial migration (Fig. 1), and increasing general interest in rapid eco‐evolutionary responses to environmental change and resulting ‘evolutionary rescue’ (e.g. Pelletier *et al.,* 2009; Bonte *et al.,* 2012; Gonzalez *et al.,* 2013; Chevin & Hoffmann, 2017), we still lack any general understanding of key forms of spatio‐temporal demographic variation, covariation, structure and heterogeneity that can arise in partially migratory systems, and of the short‐term and longer‐term population dynamic and evolutionary consequences.

## III. BASIC SCENARIOS AND MODELS OF PARTIAL MIGRATION

**Figure 2:**
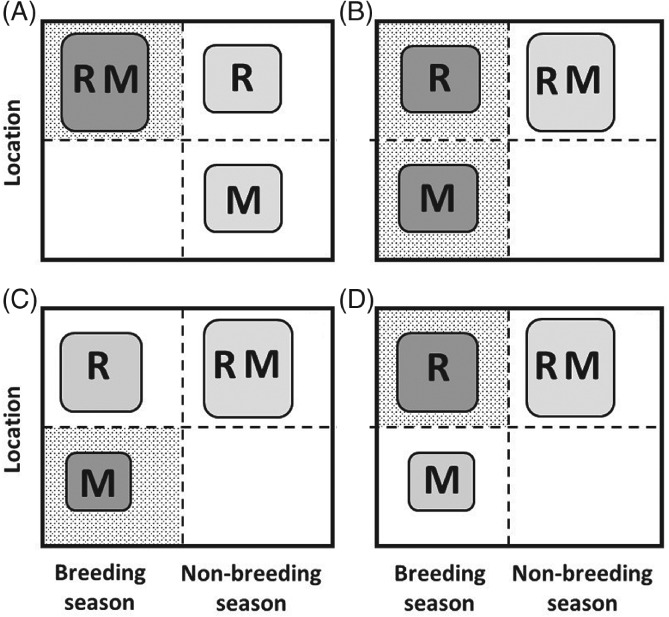
Illustration of four basic scenarios of partial migration considered as mutually exclusive alternatives. Resident (R) and migrant (M) individuals can (A) co‐exist in the same location in the breeding season but be spatially separated in the non‐breeding season (‘non‐breeding partial migration’, also known as ‘shared breeding partial migration’); or (B) co‐exist in the non‐breeding season but be spatially separated in the breeding season (‘breeding partial migration’, also known as ‘shared non‐breeding partial migration’); or (C) all individuals typically inhabit a non‐breeding location but some individuals sporadically migrate to breed at a different location during the breeding season while other individuals remain resident and hence do not breed (‘intermittent breeding partial migration’, also known as ‘skipped breeding partial migration’); or (D) all individuals typically inhabit a breeding location but some individuals sporadically migrate to a non‐breeding location during the breeding season and hence do not breed (‘intermittent non‐breeding partial migration’). Box sizes indicate local seasonal population densities, implying that density is highest when residents and migrants coexist. Background stippling indicates location–seasons where breeding can occur. Dark‐grey and light‐grey shading respectively indicate sets of individuals that do and do not breed in each season. These scenarios implicitly assume local strong seasonality such that: the migrants' non‐breeding‐season location cannot support breeding (A); the migrants' breeding location cannot support non‐breeding‐season survival (B, C); and the migrants' breeding‐season location cannot support breeding (D). Scenario C also requires an initial movement of offspring from the breeding location to the non‐breeding location.

To date, models aiming to elucidate dynamics arising in partially migratory systems have typically envisaged highly stylised two‐location systems where focal sets of resident and migrant individuals co‐exist either in the breeding season, or in the non‐breeding season, but not both. Two primary scenarios of partial migration can then be conceptualised: ‘non‐breeding partial migration’ occurs when residents and migrants co‐exist in the breeding season and are spatially separated in the non‐breeding season, and ‘breeding partial migration’ occurs when residents and migrants co‐exist in the non‐breeding season and are spatially separated in the breeding season (Fig. 2A, B; e.g. Kaitala, Kaitala, & Lundberg, 1993; Kokko & Lundberg, 2001; Taylor & Norris, 2007; Griswold, Taylor, & Norris, 2010; Griswold *et al.,* 2011; Chapman *et al.,* 2011). Elements of both scenarios are widely observed in nature. For example, instances of breeding‐season sympatry between residents and migrants occur in blackbirds (*Turdus merula*) (Fudickar *et al.,* 2013; Zúñiga *et al.,* 2017) and red‐spotted newts (*Notophthalmus viridescens*) (Grayson, Bailey, & Wilbur, 2011), while instances of non‐breeding‐season sympatry occur in American dippers (*Cinclus mexicanus*) (Gillis *et al.,* 2008) and ungulates that express partial altitudinal migration (e.g. Hebblewhite & Merrill, 2011).

A third partial migration scenario has been envisaged, where all individuals typically inhabit a non‐breeding location and some individuals sporadically migrate to a different location to breed but remain in the non‐breeding location, and hence skip reproduction, in other years (‘intermittent breeding partial migration’, Fig. 2C; Shaw & Levin, 2011). We additionally highlight a fourth scenario, where all individuals typically inhabit a breeding location but some non‐breeding individuals (e.g. sub‐adults or adults that skip reproduction) migrate to a non‐breeding location for one or multiple breeding seasons (‘intermittent non‐breeding partial migration’, Fig. 2D). This scenario could arise when breeding areas become unfavourable for non‐breeders during the breeding season, for example due to high combined densities of breeders and new offspring and associated competition for resources or risk of disease or predator attraction, but breeders cannot leave due to their reproductive requirements (e.g. breeding sites or immobile offspring). Such partial migration away from breeding locations (i.e. ‘temporary emigration’) occurs in diverse species, causing heterogeneity in local seasonal presence and associated encounter probability (for example, as highlighted in diverse marine mammals, reptiles and birds; Fujiwara & Caswell, 2002; Stauffer, Rotella, & Garrott, 2013; Weimerskirch *et al.,* 2015).

Mathematical models have been derived and analysed to examine the relative and overall population dynamics of residents and migrants within simple two‐location ‘breeding’, ‘non‐breeding’ and ‘intermittent‐breeding’ partial‐migration scenarios considered as discrete mutually exclusive alternatives [Fig. 2A–C; e.g. Kaitala *et al.,* 1993; Taylor & Norris, 2007; Griswold *et al.,* 2010, 2011; Kokko, 2011; Shaw & Levin, 2011; De Leenheer *et al.,* 2017; the ‘intermittent non‐breeding’ scenario (Fig. 2D) has not received analogous treatment]. The primary aim of such models has been to identify conditions where partial migration (i.e. migratory polymorphism) is maintained given different forms of differential reproduction and/or survival between migrants and residents and of season‐specific density‐dependence (Lundberg, 2013). Underlying variation in location‐specific seasonal density emerges as a simple function of total population size and migration rate, creating intrinsic negative frequency‐dependence in the benefit of migration and thereby maintaining migratory polymorphism. Such models predict that the evolutionary stability and degree of partial migration (as opposed to full obligate migration or residence) can depend on the relative reproductive success of migrants and residents (e.g. Kaitala *et al.,* 1993; Kokko & Lundberg, 2001), on the magnitudes of seasonal density‐dependence in survival (e.g. Taylor & Norris, 2007; Griswold *et al.,* 2010) and fertility (De Leenheer *et al.,* 2017), and on the form of asymmetry in territory acquisition (e.g. Kokko, 2011). Collectively, they highlight that the conditions that maintain partial migration differ between scenarios where migrants and residents co‐exist in the breeding *versus* non‐breeding seasons *versus* intermittent breeding, and depend on the forms of season‐specific demographic variation and associated density‐dependence and frequency‐dependence (Griswold *et al.,* 2010; Chapman *et al.,* 2011; Kokko, 2011; Shaw & Levin, 2011). They also illustrate that the overall size and composition of partially migratory populations can change, sometimes in counter‐intuitive ways, in response to environmental changes that affect breeding and/or non‐breeding locations and associated demographic rates (Griswold *et al.,* 2011; Kokko, 2011). They thereby further illustrate the role of seasonal demography in shaping population dynamics.

Such models, analyses and conclusions have been central to developing and testing theory regarding the maintenance of partial migration. However, they necessarily consider simple abstract scenarios of partial migration and invoke a key assumption that migrants and residents co‐exist in one biological season but are completely spatially separated in the other season (Fig. 2). Locations that are seasonally occupied only by focal migrants are assumed not to contain any other resident (or migrant) conspecifics, meaning that local density and consequent density‐dependent reductions in reproduction or survival arise solely from the number of migrants originating from the focal shared location (generating direct negative frequency‐dependence). Since residents and migrants do not co‐exist in both the breeding and non‐breeding seasons within any individual scenario, any demographic effects of season‐specific environments and/or density will differentially impact migrants *versus* residents, causing strategy‐specific dynamics. Further, some models assume no density‐dependent constraints on demography in a non‐shared season or location (e.g. Kaitala *et al.,* 1993; Griswold *et al.,* 2010), and emphasise seasonal release from density‐dependence as a key process that can cause partially migratory population sizes to exceed those of otherwise analogous fully resident or fully migratory populations (Griswold *et al.,* 2011).

By contrast, in real‐world partially migratory systems, single focal sets of migrants and residents are unlikely to exist in complete seasonal isolation from other sets of individuals, as envisaged in the four basic abstract scenarios (Fig. 2). Rather, given weaker environmental seasonality, migrants might often move between locations that hold different sets of residents and/or migrants originating from elsewhere. Different sets of migrants and residents can then co‐exist at different locations across breeding and non‐breeding seasons, and hence experience common environments and population densities. The conclusions of simple two‐location partial‐migration models that assume local strong seasonality, and hence complete seasonal segregation of residents and migrants, seem unlikely to hold given such generalised conditions (Holt & Fryxell, 2011).

Further, existing partial‐migration models are (deliberately) demographically and ecologically simplistic. They typically do not consider sex‐, age‐, stage‐ or state‐specific migration *versus* residence (but see Kaitala *et al.,* 1993; Kokko, 2011), or hence consider resulting spatio‐temporal variation in local sex ratios or age or stage structures. They do not consider environmentally induced plasticity, canalisation, carry‐over effects or cohort effects in migration *versus* residence itself (as opposed to specific short‐term carry‐over effects of migration on reproduction or survival). Consequently, such models have not yet encompassed forms of spatial, temporal and individual variation in life history and resulting demography that have proved necessary to adequately understand and forecast the dynamics of non‐migratory populations (e.g. Coulson *et al.,* 2001; Clutton‐Brock & Coulson, 2002; Lindström & Kokko, 2002; Benton *et al.,* 2006; Harrison *et al.,* 2011; Legrand *et al.,* 2017), and been strongly advocated for fully migratory populations (Runge & Marra, 2005; Hostetler *et al.,* 2015). Consequently, as yet, we have no overarching conceptual frameworks with which to explore, rationalise or forecast the dynamics of partially migratory populations inhabiting complex seasonally varying environments where the potential for spatio‐temporal demographic structuring, and hence for complex eco‐evolutionary responses to spatio‐temporal environmental change, is likely to be substantial.

## IV. CONCEPT AND PROPERTIES OF A ‘PARTIALLY MIGRATORY META‐POPULATION’

### (1) A partially migratory meta‐population

**Figure 3:**
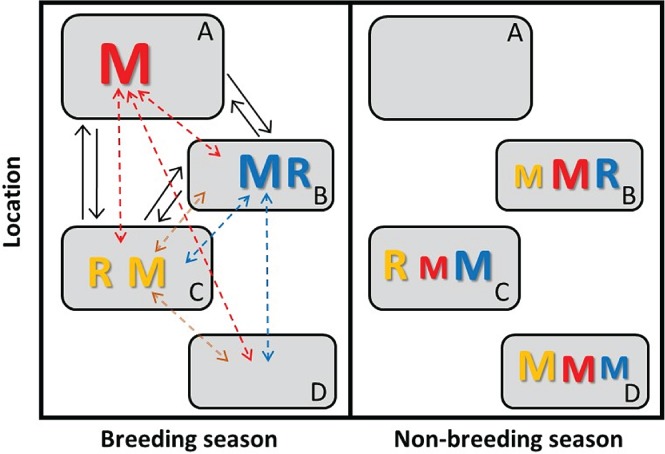
Illustration of a simple partially migratory meta‐population (PMMP) system comprising four patches, where three patches (A–C) can support breeding‐season survival and reproduction and three patches (B–D) can support non‐breeding‐season survival. Here, individuals that breed in patch B (blue font) can remain resident (R) throughout both breeding and non‐breeding seasons or migrate (M, dashed arrows) to patches C or D for the non‐breeding season. Likewise, individuals that breed in patch C (yellow font) can remain resident or migrate to patches B or D for the non‐breeding season. Individuals that breed in patch A (red font) must migrate to patches B–D for the non‐breeding season. Migration could be bidirectional (e.g. patch C to patches B and D), or reciprocal (e.g. in both directions between patches B and C), but asymmetric (font sizes denote relative numbers of individuals). Patches B and C can consequently hold different combinations of resident and migrant individuals in both seasons (left *versus* right panels). Meanwhile, patches A and D are unoccupied in the non‐breeding and breeding seasons, respectively (i.e. local populations go seasonally extinct), yet support migrants in the opposite seasons. Patches A–C that can support breeding can also be linked by dispersal (solid black arrows). This general PMMP system thereby comprises a set of locations experiencing spatio‐temporal seasonal environmental variation that can be occupied by different sets of resident, migrant and dispersed individuals in different seasons.

To provide an overarching conceptual framework for exploring the spatio‐temporal dynamics of populations inhabiting spatially structured seasonally varying environments we define a ‘partially migratory meta‐population’ (PMMP) system as a set of locations (e.g. habitat patches) that can hold sub‐populations of individuals comprising different sets of co‐existing residents and/or migrants of different sexes, ages, stages or states in different seasons (Fig. 3).

Such locations could comprise patches of similar or radically different habitats that could be immediately adjacent, forming a local habitat mosaic, or could be separated by substantial geographical or altitudinal distances. Seasonality could be strong, such that single locations cannot support both breeding‐season activity (i.e. reproduction and survival) and non‐breeding‐season activity (i.e. survival), or could be weaker such that some or all locations can support all year‐round activities to different degrees. Consequently, at the extremes, some locations could be repeatedly vacated and re‐colonised across consecutive seasons, representing spatially restricted occurrences of ‘non‐breeding’ and ‘breeding’ partial migration (Fig. 2A, B) nested within a more complex overall system (Fig. 3). However, given multiple locations and weaker seasonality, focal sets of seasonal migrants originating from any particular location could coexist with different sets of residents, and with incoming migrants originating from other locations, in both seasons (Fig. 3). Meanwhile, focal sets of residents could co‐exist with different sets of incoming migrants in both seasons (Fig. 3). The resulting year‐round local co‐existence of different sets of residents and migrants is not possible given the basic two‐location partial‐migration scenarios that have previously been conceptualised and analysed as discrete alternatives (Fig. 2).

Within a PMMP, migration could be bidirectional from individual locations, or even reciprocal among locations that can support both breeding‐season and non‐breeding‐season activity, rather than uniformly directional (Fig. 3). However, relative migration rates could be asymmetric, reflecting underlying seasonal source–sink dynamics (Fig. 3). Further, there might be spatial variation in the demographic structure of migration, for example because the sex, age, stage or state structure of migration or residence [including ‘intermittent breeding’ and ‘intermittent non‐breeding’ partial migration expressed by breeders *versus* non‐breeders (Fig. 2C, D)] varies among locations. Finally, locations that can support reproduction might also be linked by dispersal (Fig. 3), creating further demographic and genetic connectivity.

Such PMMP structures could encompass any number of locations with different relative and absolute abilities to support reproduction *versus* breeding‐season and non‐breeding‐season survival, meaning that not all conceivable seasonal transitions among locations will necessarily arise in all systems. The PMMP framework could be further extended to consider more complex forms of seasonality, encompassing systems where mating and offspring rearing are spatially separated, and incorporating additional locations used solely as migratory stop‐overs or as ‘stepping stones’ in extended spatially structured migrations (e.g. Faaborg *et al.,* 2010; Hostetler *et al.,* 2015; Thorup *et al.,* 2017). PMMP structures could therefore apply to diverse systems spanning diverse spatial scales, for example including fish inhabiting lake–stream or stream–ocean systems or patchy oceanic or estuarine environments (e.g. Kerr & Secor, 2012; Papastamatiou *et al.,* 2013; Vélez‐Espino, McLaughlin & Robillard, 2013; Hodge, Wilzbach, & Duffy, 2014), newts inhabiting pond–forest mosaics (e.g. Grayson *et al.,* 2011), and birds, mammals and reptiles inhabiting locations spanning altitudinal, latitudinal or environmental gradients (e.g. McDevitt *et al.,* 2009; Mysterud *et al.,* 2011; Fudickar *et al.,* 2013; Avgar *et al.,* 2014; Anderson *et al.,* 2015; Boyle, 2017; Grist *et al.,* 2017; Peters *et al.,* 2017; Yackulic, Blake, & Bastille‐Rousseau, 2017; Zúñiga *et al.,* 2017).

### (2) ‘Partially migratory meta‐population’ as an overarching conceptual framework

Overall, the PMMP concept provides an overarching general framework that encompasses multiple established paradigms in population ecology as special cases. First, the case where all patches can support year‐round activity and all individuals are non‐migratory represents a classical meta‐population of patches linked by dispersal, as considered by existing stochastic patch‐occupancy models and spatially explicit matrix and individual‐based models (Section I.2). Second, the case where all locations exhibit strong seasonality and all individuals migrate (or die) represents full obligate directional migration to single or multiple destinations, as considered by existing meta‐population, network and full annual cycle models of fully migratory populations (Section I.3). Third, cases where some locations can support breeding‐season but not non‐breeding‐season activity, or *vice versa* (i.e. local strong seasonality), can reduce to the basic discrete two‐location scenarios considered by existing partial‐migration models (Section III).

In comparison, the full general PMMP concept (Fig. 3) allows greater spatio‐temporal variation in the degree of environmental seasonality, and such landscapes could consequently support spatially, temporally and demographically diverse sets of interacting residents and seasonal migrants. PMMPs could thereby create diverse and complex forms of spatio‐temporal variation, covariation, structure and heterogeneity in and among individual reproduction, survival, dispersal and migration, including complex forms of migratory plasticity, carry‐over effects and density‐dependence and hence migratory frequency‐dependence. Such PMMP structures might consequently create substantially different population dynamic and evolutionary responses to seasonal environmental variation, including extreme seasonal environmental events, from those arising in non‐migratory or fully migratory populations or given basic partial‐migration scenarios, affecting the locations, seasons and sub‐populations that underpin population persistence. Numerous effects that encompass structured demographic variation and potentially rapid evolution can be hypothesised, as outlined in the following sections and summarised in Tables 1 and 2.

**Table 1:** Three sets of key conceptual and theoretical questions that need to be addressed in order to understand and forecast population and evolutionary dynamics in partially migratory meta‐populations (PMMPs) in seasonally varying environments.

**(1) Prospective analyses of population growth rate** (*i*) What are the sensitivities (or elasticities) of sub‐population and overall PMMP growth rates to sex‐, age‐ and sub‐population‐specific migration rates, and to covariances among migration *versus* residence and survival, dispersal and reproduction? (*ii*) What are the sensitivities (or elasticities) of sub‐population and overall PMMP growth rates to the degree of individual migratory plasticity and associated carry‐over effects? (*iii*) How do such sensitivities (or elasticities) vary with life history (i.e. with mean demographic rates), and with the spatial structures of seasonal environmental variation and density‐dependence and resulting migratory frequency‐dependence?
**(2) Complex population dynamics and persistence given environmental change** (*i*) What are the responses of sub‐population and overall PMMP growth rates and extinction probabilities to postulated regimes of seasonal environmental change, including extreme environmental events and resulting major demographic perturbations? What are the resulting rates of seasonal range‐shifting, and the time courses and spatial scales of internal and overall transient and cyclic population dynamics? (*ii*) How do such spatio‐temporal dynamics vary with life history, with the form of individual migratory plasticity, with the spatial structure of seasonal environmental variation and change, and with the spatio‐temporal pattern of major perturbations? (*iii*) To what degree are PMMP dynamics and local and global extinction probabilities more or less resilient or responsive to seasonal environmental change and perturbations than classical meta‐populations (i.e. spatially structured populations with dispersal but no seasonal migration), or than populations with full obligate migration?
**(3) Genetic variation and eco‐evolutionary dynamics** (*i*) To what degree can PMMP structures maintain additive genetic variation in individual liability (i.e. propensity) for migration *versus* residence, including through periods of environmental change and range‐shifting? How does such maintenance vary with life history, and with the spatial structures of seasonal environmental variation and of major perturbations? (*ii*) How does the evolved strategy of migration *versus* residence, and the magnitude of additive genetic variation, vary among PMMP sub‐populations, thereby generating local adaptation and evolvability in migration? How do such properties vary with the spatial structure of seasonal environmental variation and perturbations? (*iii*) To what degree can PMMP structures facilitate or impede evolution of phenotypic plasticity in the form of migration *versus* residence, fostering condition‐dependence and responsive ‘irruptive’ migratory responses to extreme environmental events? (*iv*) To what degree can evolution of migration facilitate or impede evolution of dispersal (and *vice versa*)? What are the directions and magnitudes of emerging genetic covariances? (v) To what degree do such evolutionary dynamics feed back to affect PMMP dynamics and persistence, and over what time scales? Consequently, to what degree can changing partial migration generate rapid ‘evolutionary rescue’ in the face of seasonal environmental change, including major perturbations?

**Table 2:** Three sets of key empirical questions that need to be addressed in diverse partially migratory populations and meta‐populations (PMMPs) in order to inform, parameterise, test and/or validate population and evolutionary dynamic theory and models.

**1. Spatial and demographic structures of partial migration, including individual canalisation and plasticity** (*i*) To what degree does the form of migration or residence differ between females and males of different ages, stages or states originating from the same and different locations within PMMPs? Consequently, what is the form of structured partial migration, and resulting seasonal co‐existence of different types of residents and migrants, across different locations? (*ii*) How does the form of migration or residence expressed by individuals develop across ages and life‐history stages? Consequently, is the form of migration or residence canalised or plastic within or across ontogenic stages? (*iii*) To what degree is the form of migration or residence expressed by individuals canalised or plastic across different environmental conditions? Specifically, what are the forms of environment‐dependence and/or density‐dependence in migration or residence? (*iv*) To what degree do initial environment‐dependent and/or density‐dependent migration and developmental canalisation combine to generate persistent cohort effects in the form of migration or residence? (*v*) To what degree do atypical extreme environmental events increase or decrease switching between residence and migration (for example causing responsive irruptive migration)? Does such responsive migration have carry‐over effects on subsequent expression of pre‐emptive migration? (*vi*) To what degree does the magnitude or form of plasticity in migration expressed in relation to typical or atypical ranges of seasonal environmental variation or density vary among sexes, age or stage classes, cohorts or locations?
**(2) Key life‐history and demographic covariances involving partial migration** (*i*) To what degree do survival, reproduction and dispersal vary with different forms of migration or residence, including direct effects and carry‐over (lagged) effects? Conversely, to what degree does the form of migration or residence vary in response to reproduction and dispersal? (*ii*) To what degree do such direct and lagged demographic covariances vary with sex, age, stage or state, thereby defining the overall structure of demographic covariation? (*iii*) To what degree do such direct and lagged demographic covariances vary among individuals that inhabit different locations and hence experience different seasonal environments and degrees of environmental seasonality, thereby defining the spatial structure of demographic covariation? (*iv*) To what degree does such spatial variation in demographic covariance vary temporally, in relation to typical or atypical seasonal environmental variation, thereby defining the spatio‐temporal structure of demographic covariation? (*v*) To what degree are effects of migration *versus* residence on survival, reproduction and dispersal manifested among breeding pairs or groups rather than solely among individuals?
**(3) Key genetic variances and covariances involving partial migration** (*i*) What is the magnitude of additive genetic variance in individual liability (i.e. propensity) for migration *versus* residence? What are the magnitudes of other components of phenotypic variance, for example individual, year, cohort and parental environmental variances? Hence what is the narrow‐sense heritability of migration manifested in the context of natural environmental variation and hence total phenotypic variance? (*ii*) What is the magnitude of additive genetic variance in migratory plasticity? (*iii*) What are the additive genetic covariances between liability for migration and survival, reproduction and dispersal, and hence what are the forms of evolutionary drivers and constraints? (*iv*) To what degree do these additive genetic variances and covariances differ between females and males, and vary among sub‐populations originating from or inhabiting different locations? (*v*) What is the degree of assortative mating between different sets of migrants and residents? How does the degree of assortative mating vary among stages, years and locations?

#### (a) *Partial migration, reproduction and survival*

**Figure 4:**
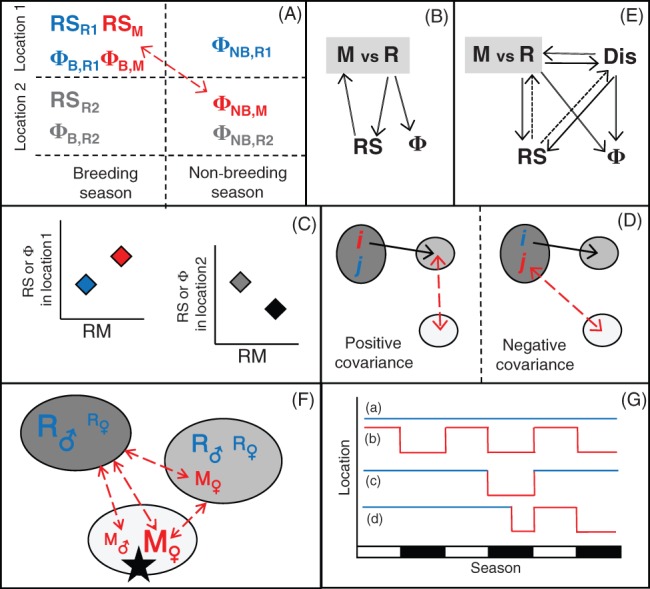
Illustrations of key forms of demographic structure and covariation that could arise in partially migratory meta‐populations. (A) Migrants that breed in location 1 and spend the non‐breeding season in location 2 (red font) could have different reproductive success (RS_M_) and breeding‐season survival (Φ_B,M_) from seasonally sympatric residents in location 1 (blue font, RS_R1_, Φ_B,R1_), and from seasonally allopatric residents in location 2 (grey font, RS_R2_, Φ_B,R2_). These migrants could then have different non‐breeding‐season survival (Φ_NB,M_) from seasonally sympatric residents in location 2 (Φ_NB,R2_), and from seasonally allopatric residents in location 1 (Φ_NB,R1_), creating additional structure in key demographic rates in both seasons. (B) An individual's form of migration *versus* residence (M *vs* R) might affect its reproductive success (RS), which might feed back to affect its subsequent migration or residence and resulting survival (Φ). (C) Migrants (M) that breed in location 1 might have higher reproductive success or seasonal survival than local residents (R), while migrants that breed in location 2 might have lower reproductive success or seasonal survival than local residents, creating spatially disruptive selection on migration. (D) Covariances between dispersal and migration. Covariance could be positive (left panel), where individuals *i* that disperse (solid arrow) from their natal location (dark grey) to a different breeding location (mid grey) are more likely to migrate (red dashed arrow) to a different non‐breeding‐season location (light grey) than individuals *j* that do not disperse. Conversely, covariance could be negative (right panel), where individuals *i* that disperse from their natal location are less likely to migrate than individuals *j* that do not disperse. (E) Complex feedbacks: the forms of migration *versus* residence (M vs R) and dispersal (Dis) could affect RS and Φ directly, and also indirectly if they affect each other (double arrows). Resulting RS could then feed back to affect the form of subsequent migration *versus* residence and dispersal (dotted arrows). (F) Example of non‐breeding‐season demographic structure arising if females are more likely to migrate (red font) from breeding areas (dark grey) to different non‐breeding areas (light grey) than males, while males are more likely to remain resident (blue font). Font sizes denote relative frequencies. An extreme environmental event in the non‐breeding area (black star) would then disproportionately impact migrant females. (G) Individual (a) residence (blue) or (b) migration (red) could be fixed and consistently expressed across multiple breeding and non‐breeding seasons, or could be plastic and expressed either (c) pre‐emptively or (d) responsively in some seasons but not others. Responsive migration in one season might lead to future pre‐emptive migration (d).

Most obviously, variation in the occurrence of individual migration *versus* residence within and/or across locations could directly cause substantial within‐ and among‐location variation in individuals' survival and/or current or subsequent reproduction. Migrants inhabiting different locations in different seasons could be more or less likely to survive and/or reproduce than residents in the same sex, age or stage classes inhabiting the same initial or destination locations, creating major demographic structure among different sets of individuals that co‐exist in one season or the other (Fig. 4A). Such effects could stem from major physiological changes associated with the migratory movement itself, and/or from different environmental, information or social conditions experienced as a consequence of migration. Indeed, individuals migrating within PMMPs might experience competition with established local residents in both seasons (e.g. Fig. 4A), rather than in only one season as assumed in models that consider basic two‐location partial‐migration scenarios (e.g. Taylor & Norris, 2007; Kokko, 2011, Fig. 2), or in neither season as assumed in models of fully migratory populations (e.g. Runge & Marra, 2005; see also Kokko *et al.,* 2006). Such effects could exacerbate socially induced costs and constraints on migration or residence (e.g. Pérez‐Tris & Tellería, 2002; Mysterud *et al.,* 2011; Cote *et al.,* 2017).

More complex carry‐over effects could arise when migration substantially affects multiple future life‐history traits expressed by individuals, including future migration itself. For example, migration *versus* residence might facilitate or impede subsequent reproduction, which might alter future expression of migration or residence, thereby affecting subsequent survival or reproduction (Fig. 4B). Indeed, in partially migratory Cory's shearwaters (*Calonectris borealis*), experimentally induced breeding failure caused reduced migration in males, followed by earlier return to the breeding colony and higher breeding probability the following year (Catry *et al.,* 2013). Cross‐season associations between individual breeding failure and non‐breeding‐season location have also been observed in fully migratory black‐legged kittiwakes (*Rissa tridactyla*; Bogdanova *et al.,* 2011).

Further, within a PMMP, direct and lagged effects of the expression of migration or residence on survival and reproduction are themselves likely to be spatially heterogeneous (Fig. 4C). This is because seasonal environmental conditions, and hence the magnitude of local environmental seasonality, will likely vary spatially. Consequently, migration might increase survival and reproductive success in some sub‐populations if migrants experience better seasonal conditions, but decrease survival and reproductive success in other sub‐populations if any survival benefit of migration is outweighed by associated costs, and/or migrants lose breeding opportunities to local residents (Fig. 4C). Such reversed effects of migration *versus* residence on survival and reproduction across locations would generate system‐wide disruptive selection, potentially leading to local migratory adaptation.

#### (b) *Partial migration and dispersal*

The existence of multiple locations with relatively weak seasonality means that covariances between occurrences of migration and dispersal might also arise. Individuals that disperse to a non‐natal breeding location might be more (or less) likely to undertake subsequent seasonal migrations than non‐dispersers inhabiting the same breeding and/or natal locations (Fig. 4D). Similarly, individuals that migrate might be more (or less) likely to subsequently disperse (Fig. 4D). Positive covariances could arise if the same underlying physiology or ecological drivers facilitate both forms of movement (e.g. Cote *et al.,* 2017), or because dispersers gain wider spatial environmental experience than non‐dispersers, thereby facilitating future informed migration (or *vice versa*). Conversely, negative covariances could arise if, by dispersing, individuals can move to a good year‐round location and thereby eliminate the need for subsequent migration. Indeed, an initial migratory movement with failure to return (whether reflecting adaptive decision or constraint) equates to dispersal (as currently defined; Berthold, 1999; Cote *et al.,* 2017, see also Section V.2).

Direct effects of migration on survival and reproduction could then occur non‐independently of direct negative or positive effects of dispersal. Such effects are themselves widely considered to be multifaceted and substantial, representing multiple costs of dispersal alongside benefits stemming from release from high local density and kin competition (Bonte *et al.,* 2012). Migration and dispersal could then have further indirect effects on survival and reproduction because they affect each other (Fig. 4E). Additional carry‐over effects of reproduction on migration could then arise if reproductive failure prompts dispersal (e.g. Hoover, 2003) and dispersal affects an individual's subsequent migration (Fig. 4E).

Overall, PMMP structures could therefore magnify or mitigate the independent consequences of seasonal migration and dispersal for reproduction and survival, and hence further affect population and evolutionary dynamics. Introgressive gene‐flow stemming from dispersal and subsequent reproduction might then be non‐independent of transient (non‐introgressive) gene flow stemming from seasonal migration, further shaping the overall evolutionary and population consequences of spatio‐temporal variation in seasonal selection.

#### (c) *Partial migration and density*

Patterns of spatio‐temporal variation in population density arising in PMMPs, and hence expression of density‐dependence in reproduction, survival, dispersal and migration (and resulting frequency‐dependent consequences of migration and dispersal), are likely to differ substantially from those arising in non‐migratory or fully migratory populations, or given basic two‐location partial‐migration scenarios (Section III). In a PMMP, local seasonal density is no longer a simple function of total population size in the preceding season and local migration rate. Rather, local seasonal densities will result from variation in residence, migration and dispersal of individuals originating from, and potentially moving to, numerous initial and destination locations, experiencing different seasonalities and hence forms of selection on migration and dispersal (Fig. 3). Indirect forms of lagged density‐ and frequency‐dependence could also arise if the presence of residents or other migrants in a location in the season when a focal set of migrants is absent alters the conditions the focal migrants experience upon return, for example by depleting food resources or maintaining predator or parasite populations.

#### (d) *Structure, heterogeneity and individual variation in migration versus residence*

The overall forms and magnitudes of demographic structure and heterogeneity arising within a PMMP could be further complicated and exacerbated if variation in migration *versus* residence is itself strongly structured across different sets of individuals. As with any life‐history trait, the occurrence and form of migration might vary with sex, age, stage or state, such that particular classes of individuals are more likely to remain resident *versus* migrate from or to particular locations (Fig. 4F). Such effects could arise if different classes have different seasonal resource requirements or resilience to seasonal environments, or different dominance in competition for seasonally restricted resources (e.g. Jahn *et al.,* 2010; Chapman *et al.,* 2011; Grayson *et al.,* 2011; Kokko, 2011; Fudickar *et al.,* 2013; Avgar *et al.,* 2014; Yackulic *et al.,* 2017). The degrees of sex‐, age‐, stage‐ or state‐specific residence and outgoing and incoming migration are then likely to vary among locations within a PMMP, depending on the degrees of local environmental seasonality and population density, which itself stems from the sum total of local residents and incoming and outgoing migrants (Fig. 4F). Residents and migrants that co‐exist in particular locations in specific seasons could consequently be similar or different in sex, age, stage or state, creating substantial spatial and seasonal heterogeneity in sub‐population composition (Fig. 4F).

Such season‐specific and location‐specific demographic structure in turn increases the risk that particular sets of migrants and residents will be disproportionately affected by spatially restricted extreme seasonal environmental events (e.g. extreme weather, disease, predation, pollution or habitat destruction; Fig. 4F). Events that cause high mortality in particular locations in particular seasons could then create spatially dynamic sex ratios and age or stage structures that could affect future reproductive opportunities stemming from local density and mate availability, and hence drive subsequent dispersal across numerous other locations. In a PMMP, such episodes would also generate strong sex‐, age‐ or stage‐specific selection on the expression of migration *versus* residence, potentially driving further evolution of structured partial migration.

Indeed, PMMPs might show complex responses to any local extreme event and resulting demographic perturbation. They might show rapid internal dynamics but damped overall dynamics if individuals can escape extreme local events by temporarily changing location by facultatively switching from residence to migration (i.e. rapid plasticity, generating ‘irruptive’ migration that is environmentally responsive rather than pre‐emptive; Fig. 4G). Conversely, they might show lagged internal dynamics and exacerbated overall dynamics if individual migration or residence is largely genetically determined or strongly environmentally canalised and hence remains fixed through all circumstances (Fig. 4G). Extreme events that directly impact some locations could then cause major cross‐season demographic perturbations in other locations, potentially reshaping population composition and structure across spatio‐temporal scales that greatly exceed the original extreme event. Such dynamics could be further exacerbated if there are additional carry‐over or ‘learned’ effects of facultative irruptive migration, such that surviving individuals or their offspring are more (or less) likely to pre‐emptively migrate in subsequent years (Fig. 4G). Further, state‐, environment‐ and density‐dependent effects on migration could be expressed over multiple years, for example if individuals' initial strategies of migration or residence are affected by natal conditions and then become canalised, generating temporally persistent but spatially varying cohort effects in migration.

#### (d) *Eco‐evolutionary dynamics involving partial migration*

Divergent associations between the form of migration *versus* residence and reproduction and survival expressed across different sex, age or stage classes and location–seasons will shape the forms of spatio‐temporal variation in selection on migration, and the resulting evolution of sex‐, stage‐, state‐ or location‐specific migration propensity and plasticity (given underlying additive genetic variation). Spatially disruptive selection arising within a PMMP (e.g. Fig. 4C) could help maintain system‐wide genetic variation in migration, and thereby maintain a major life‐history polymorphism that creates substantial seasonal demographic structuring yet potentially facilitates future rapid evolutionary switches towards full population‐wide residence or migration given further environmental change (e.g. Roff, 1996; Pulido, 2007, 2011; McGuigan & Sgrò, 2009; Pulido & Berthold, 2010). Such spatial variation in selection on migration might thereby facilitate the maintenance of partial migration without necessarily requiring strong density‐dependence or resulting negative frequency‐dependence. However, surprisingly, the role of spatial variation in selection in maintaining overall partial migration, as opposed to temporal variation in selection (e.g. Kaitala *et al.,* 1993; Lundberg, 2013), has scarcely been considered. Variation in other life‐history traits that are genetically correlated with individual propensity for migration, potentially including propensities for dispersal, reproduction and survival, might consequently also be maintained (e.g. Quinn, Unwin, & Kinnison, 2000).

Further, associations between the form of migration *versus* residence and key aspects of reproductive activity, such as reproductive timing, could potentially generate assortative mating within sets of migrants and residents that breed in any particular location (Anderson *et al.,* 2015). Such assortative mating could cause cryptic genetic structuring and reproductive isolation within seasonally sympatric sub‐populations (as observed across migratory divides; e.g. Bearhop *et al.,* 2005; Liedvogel *et al.,* 2011). It could simultaneously magnify heterogeneity in reproductive success, for example between migrant–migrant *versus* resident–resident pairings, and thereby magnify selection differentials on migration (e.g. Grist *et al.,* 2017). However if selection on migration varies spatially, as is likely when the degree of environmental seasonality varies among locations (Fig. 4C), then dispersal and resulting introgressive gene flow could prevent evolution of locally adaptive strategies of migration or residence. Such systems might consequently foster evolution of increased phenotypic plasticity in migration, and hence increased individual migratory responsiveness to local environmental conditions. Extreme environmental events and resulting facultative expression of irruptive migration (e.g. Fig. 4G) might then expose otherwise hidden genetic variation to selection (e.g. McGuigan & Sgrò, 2009) and facilitate or impede further expression or evolution of individual plasticity (e.g. Chevin & Hoffmann, 2017).

## V. REQUIREMENTS AND OPPORTUNITIES FOR MODELLING DYNAMICS

As outlined in the preceding sections, the PMMP framework generates numerous hypotheses and questions regarding how the combination of spatio‐temporal seasonal environmental variation and structured partial migration could generate variation, covariation, structure and heterogeneity spanning all four key demographic rates, and spanning different sets of seasonally co‐existing residents and migrants (Tables 1 & 2). It thereby highlights multiple ways in which individual life‐history variation could drive complex population dynamic and evolutionary responses to spatio‐temporal environmental change. New models and empirical studies are now needed to address key hypotheses and questions regarding PMMP demography and spatio‐temporal dynamics, to identify general principles and, ultimately, to allow forecasting for PMMP‐like systems of conservation or economic value. The requirements for modelling and empirical data might seem daunting. However, a progression from reductionist to increasingly complex models developed through multiple complementary approaches, and informed by estimates of key demographic rates and relationships from tractable empirical systems, could be used to identify processes that might generally be important *versus* ignorable, and thereby to identify priorities for further modelling and estimation.

### (1) Progression of modelling approaches

Mathematical models that consider basic partial‐migration scenarios (Section III, Fig. 2) could initially be extended to capture PMMP principles, for example by allowing some degree of reproduction and non‐breeding survival in two or more locations (i.e. weak seasonality). Indeed, Holt & Fryxell (2011) analysed a simple two‐location weak seasonality model, and showed that partial migration (i.e. migratory polymorphism) was readily maintained. Cobben & Van Noordwijk (2016) simulated a population inhabiting a landscape characterised by linear counter‐gradients of suitabilities for reproduction and non‐breeding‐season survival, and demonstrated that partial migration readily persisted within a core landscape zone given non‐zero dispersal. These two models make numerous simplifying assumptions, including no sex, age or stage structure or environmental stochasticity, no environmental state‐dependence or plasticity in migration and no carry‐over effects of migration on reproduction. Further, and importantly, they assume no direct costs of migration and either no density‐dependence in non‐breeding‐season survival (Holt & Fryxell, 2011) or constant uniform non‐breeding‐season survival of migrants with no contribution to density‐dependent survival of residents (Cobben & Van Noordwijk, 2016). They thereby impose minimal constraints on migration. These models are consequently best viewed as first steps towards future models that consider multiple key aspects of demographic variation associated with migration *versus* residence and resulting population dynamics (Holt & Fryxell, 2011; Fryxell & Holt, 2013). However, they imply that the question of how migratory polymorphism and resulting partial migration is maintained, which has been the primary focus of most previous partial‐migration models (e.g. Kaitala *et al.,* 1993; Taylor & Norris, 2007; Griswold *et al.,* 2010; Chapman *et al.,* 2011; Shaw & Levin, 2011; Lundberg, 2013), might be readily resolved when spatial variation in seasonality and dispersal are considered. New work can then shift to answering key questions regarding emerging forms of partial migration and the demographic and population dynamic consequences, including local and range‐wide dynamics, persistence and eco‐evolutionary feedbacks (Table 1).

Modelling developments could follow a similar trajectory of increasing complexity and flexibility as achieved by demographic theory and models for non‐migratory, fully migratory and dispersive systems (e.g. Caswell, 2001; Hostetler *et al.,* 2015; Lurgi *et al.,* 2015; Legrand *et al.,* 2017). Relatively simple models could initially be used to quantify sensitivities (or elasticities) of sub‐population and overall population growth rates to generalised patterns of location‐specific migration rates occurring within a PMMP, thereby characterising sub‐populations or entire PMMPs whose dynamics might be more (or less) affected by changing partial migration (Table 1). Such sensitivities are likely to vary systematically with species life history, defined by mean demographic rates including migration rate, and will also depend on aspects of PMMP spatial structure, including the degree of spatial variation in seasonality. Sensitivity analyses for PMMPs will therefore need to define dimensions of variation that quantify such structure and seasonality. This could be achieved by considering environmental gradients of varying form or magnitude (e.g. Shaw & Couzin, 2013; Cobben & Van Noordwijk, 2016), or by deriving metrics that summarise key aspects of landscape or network structure, or migratory connectivity and dispersion, analogous to existing descriptors for fully migratory systems (e.g. Taylor & Norris, 2010; Taylor & Hall, 2012; Gilroy *et al.,* 2016).

Population dynamic models could initially consider one sex and simple age or stage structure with different forms of seasonal density‐dependence. Further demographic complexity can then be added, including structural direct and lagged covariances between migration and survival, reproduction and/or dispersal, representing postulated direct costs of migration and carry‐over effects (e.g. Runge & Marra, 2005; Taylor & Norris, 2010). Sensitivities that encompass such covariances (e.g. ‘integrated elasticities’, Van Tienderen, 2000) can then be computed. Models can then be further extended to consider more complex age structure and/or two sexes and associated mating systems. Sex, age and/or stage structure in migration could be imposed, or could be allowed to emerge from underlying rules regarding state‐dependent migration (e.g. Kokko, 2011). Different degrees of developmental and environmental canalisation *versus* plasticity of migration can then be incorporated, defined by reaction norms in migratory responses to individual stage or state and local seasonal environmental conditions. Population dynamic consequences of major demographic perturbations, representing extreme seasonal environmental events that severely impact local survival or reproduction and cause irruptive (i.e. responsive) migration and associated carry‐over effects, can then be considered. Finally, eco‐evolutionary dynamics can be evaluated by considering an implicit or explicit genetic basis to migration, potentially with fixed or dynamic genetic covariances (including trade‐offs) with survival, dispersal and/or reproduction, and fixed or dynamic plasticity or canalisation.

Progress through initial objectives could be achieved using matrix models, thereby harnessing existing general methods for quantifying sensitivities and elasticities of population growth rates (Caswell, 2001). Indeed, Vélez‐Espino *et al*. (2013) derived, parameterised and analysed a matrix model for partially migratory brook trout (*Salvelinus fontinalis*) inhabiting a stream–lake system. Vélez‐Espino *et al*. (2013) defined different demographic rates for residents and migrants, implicitly assumed that individuals could switch strategies, and considered density‐dependence in juvenile survival and environmental stochasticity in fecundity. They showed that population growth rate was most sensitive to adult migrant survival, to a degree that depended on adult migration rate. Meanwhile, sensitivity to juvenile migration rate and initial survival depended on environmental conditions and associated variation in fecundity. Their analyses therefore illustrate that the growth rate and spatial structure of partially migratory populations, and elasticities of migration rate and costs, can depend on complex interactions between life history, environmental stochasticity and resulting density and hence density‐dependence expressed in the shared seasonal habitat. Similar models could potentially be parameterised and analysed for a wide range of real or hypothetical life histories, thereby yielding general patterns. However, Vélez‐Espino *et al*.'s (2013) model considers a single female‐only sub‐population with simple spatial structure and juvenile–adult stage structure, and consequently does not consider demographic variation or density‐dependence arising across multiple locations, or explicitly consider individual heterogeneity, plasticity or complex carry‐over effects.

PMMPs could also be modelled as networks comprising nodes (i.e. locations) linked by edges (i.e. routes of movement), where some or all nodes can be utilised in multiple seasons. This approach extends existing network models defined for fully migratory populations (i.e. where individual nodes can only be utilised in one season), thereby harnessing general metrics describing network connectivity and resilience (e.g. Taylor & Norris, 2010). Indeed, network models provide a flexible approach that can in principle encompass any spatial structure and form of seasonality, and hence model any desired combination of non‐migratory, fully migratory and partially migratory sub‐populations (Sample *et al.,* 2018).

However, formulating and analysing general matrix and network models that encompass spatio‐temporal seasonal variation in sex–stage‐specific demographic rates and resulting density, canalisation *versus* plasticity, and individual carry‐over effects that span multiple demographic rates, seasons and locations (e.g. Fig. 4) is likely to be challenging. Furthermore, while matrix model elasticities can be linked to selection gradients (e.g. Grant & Benton, 2000; Van Tienderen, 2000; Caswell, 2001), matrix and network models do not readily capture short‐term evolutionary dynamics of migration that could feed back to influence all key demographic rates, variances and covariances that cause population dynamics. Spatially, demographically and genetically explicit IBMs provide a means of examining context‐dependent costs and benefits and eco‐evolutionary dynamics arising in PMMPs that may be more immediately tractable despite resulting model complexity. Such models could track population dynamics and evolving strategies of migration *versus* residence both within individual sub‐populations and across overall PMMPs. Indeed, an ultimate objective should be to extend existing flexible IBM frameworks for spatially explicit population dynamic forecasting, that currently focus on dispersal as the only form of movement (e.g. Bocedi *et al.,* 2014; Lurgi *et al.,* 2015; Legrand *et al.,* 2017), to include variation in migration and associated seasonal demography. Such frameworks would, in principle, allow system‐specific forecasting of PMMP dynamics and persistence given any real or hypothetical landscape and postulated scenario of spatio‐temporal seasonal environmental change. In contrast to network models (Sample *et al.,* 2018), such IBMs do not necessarily require focal populations to inhabit landscapes where discrete habitat patches can be clearly defined.

### (2) An illustrative individual‐based model

**Figure 5:**
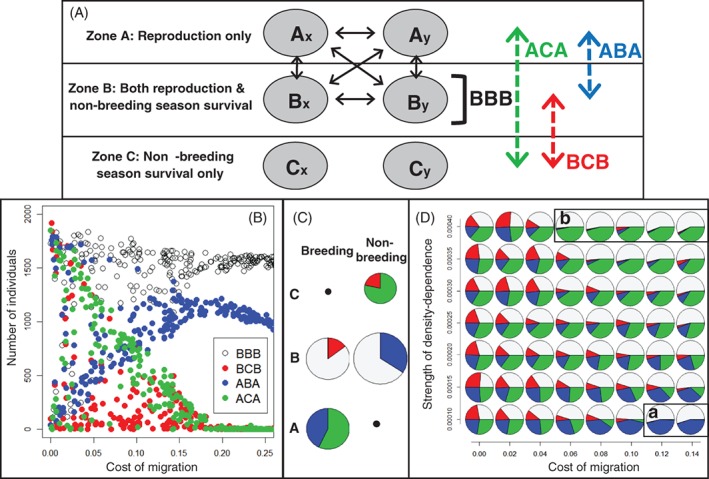
Summary of an evolutionary individual‐based model illustrating that multiple strategies of migration and residence can readily co‐exist across broad parameter space. (A) The modelled spatial structure comprised three zones that support reproduction (zone A), non‐breeding‐season survival (zone C) and both activities (zone B), with two patches (x and y) within each zone. Strategies of year‐round residence in B (BBB, black), and three forms of migration (dashed arrows: ACA, green; ABA, blue; and BCB, red), can all evolve. Dispersal can occur between x and y patches within A and B (solid arrows). (B) The number of indiviuals expressing each strategy after 20000 simulated generations of evolution varied with the survival cost of migration, but multiple strategies co‐existed over wide parameter space, generating multiple forms of partial migration. Data are from 500 independent simulations spanning a range of costs. (C) Snapshot of emerging spatio‐temporal variation in sub‐population size and composition, where pie and segment sizes respectively denote the total number of individuals, and the number of individuals expressing each strategy, that are present in the A, B, and C zones in the breeding and non‐breeding seasons. Black points indicate zone‐seasons with zero population. Example data are from one simulation, with cost of migration of 0.06 and strength of density‐dependence in non‐breeding‐season survival of 0.00015. (D) The proportion of individuals expressing each strategy after 20000 simulated generations also varied with the strength of density‐dependence in non‐breeding‐season survival, with an interaction with the survival cost of migration (probability of mortality during inter‐zone movement). Given high costs, population composition reduced to simple two‐location partial migration (segment a), or ‘leap‐frog’ migration (segment b), depending on the strength of density‐dependence. Data are from 20 replicate simulations for each combination of cost and density‐dependence. See Appendix S1 for details of the model.

To illustrate one possible approach to modelling PMMP dynamics, we built a simple IBM that tracks evolutionary dynamics of migration and dispersal, and resulting spatio‐temporal population dynamics, given spatial variation in seasonal environmental conditions and hence in non‐breeding‐season survival and subsequent reproduction. We set a simple spatial structure comprising an implicit three‐zone gradient of seasonality, where zone A can support reproduction but not non‐breeding‐season survival, zone C can support non‐breeding‐season survival but not reproduction, and zone B can support both seasonal activities (Fig. 5A). Each zone of seasonality contains two identical patches (x and y), thereby allowing dispersal within as well as between zones (Fig. 5A). This structure provides a simple extension of previous partial‐migration models that consider two locations (e.g. Fig. 2) and hence do not capture key PMMP properties (e.g. Figs 3, 4). For illustrative purposes we considered a simple haploid annual organism that is born, then moves or does not, then survives or dies during a non‐breeding season, then moves or does not, then breeds and dies. Even this simple life history and spatial structure allows multiple biologically viable individual movement strategies spanning the three considered seasons (i.e. born, non‐breeding, breeding). These comprise residence in zone B (BBB), migration between A and C (ACA), between A and B (ABA) or between B and C (BCB), and dispersal between patches within or between A and B with or without migration (Fig. 5A). Individuals' preferences for both breeding‐ and non‐breeding‐season zone occupancy, and probability of dispersing between the x and y strata, all evolve independently as determined by eight underlying genes that mutate independently. Expression of migration and dispersal incur direct costs of reduced survival, and non‐breeding‐season survival and subsequent reproduction both show negative density‐dependence, respectively specified as linear and Ricker functions of patch‐level seasonal density. Full model details are provided as online supporting information (Appendix S1).

Simulations illustrate the fundamental point that multiple different forms of partial migration are readily maintained, comprising varying frequencies of all three possible forms of migration (i.e. ABA, BCB and ACA) alongside residence (BBB, Figs 5B–D). The frequencies of individuals expressing the four different strategies varied with the survival cost of migration (Fig. 5B), to degrees that depended on the strength of density‐dependence in non‐breeding‐season survival (Fig. 5D). However, all four strategies commonly co‐existed over broad parameter space, generating substantial spatio‐temporal variation in sub‐population composition (e.g. Fig. 5C). However, given a high survival cost of migration, population composition reduced to simple two‐location partial migration given weak density‐dependence (Figs 5Da), and to ‘leap‐frog’ migration given strong density‐dependence (Figs 5Db). A low, but non‐zero, dispersal probability also systematically evolved.

**Figure 6:**
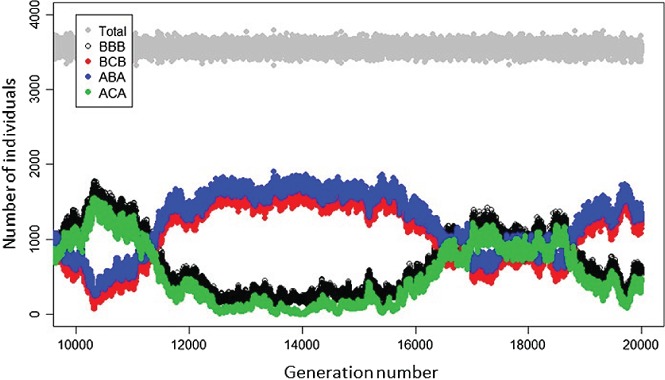
Example time series from one simulation from an evolutionary individual‐based model illustrating population dynamics of individuals enacting three strategies of migration among zones A, B and C spanning three seasons (i.e. ABA, ACA and BCB), and of lifelong residence in zone B (i.e. BBB), and of total population size across 10000 simulated generations. Zone and movement structures are shown in Fig. 5A. See Appendix S1 for details of the model.

Further examination of time series from individual simulations showed that cyclical population dynamics of individuals following complementary movement strategies arose for some parameter combinations. For example, with a small cost of migration and weak density‐dependence in non‐breeding‐season survival, total population size remained relatively constant across generations, but underlying increases in populations of ABA and BCB migrants were mirrored by corresponding decreases in populations of ACA migrants and BBB residents, and *vice versa* (Fig. 6). These two emerging pairs of spatially complementary strategies maximise space‐use in both seasons, and thereby minimise density‐dependent constraints on both non‐breeding‐season survival and subsequent reproduction.

Overall, these illustrative simulations show that even simple life histories and spatial structures and associated constraints on survival and reproduction can foster multiple co‐existing and temporally dynamic forms of partial migration and dispersal, as is increasingly widely observed in wild populations (e.g. Fig. 1; Grayson *et al.,* 2011; Singh *et al.,* 2012; Papastamatiou *et al.,* 2013; Hodge *et al.,* 2014; Anderson *et al.,* 2015). They also illustrate how simple rules regarding seasonal movement decisions can generate diverse individual strategies that span classical definitions of dispersal and migration without requiring such strategies to be explicitly specified. Such models now offer exciting opportunities to address numerous key questions concerning how different regimes of spatio‐temporal environmental variation foster different interacting strategies of migration and dispersal; to identify general rules spanning different stage‐structured life histories, spatial structures of seasonality and forms of density‐dependence, plasticity, carry‐over effects and costs; and to assess eco‐evolutionary and population dynamic consequences of diverse forms of spatio‐temporal environmental variation including local and global persistence, transient and cyclic dynamics and range‐shifting (Table 1). To achieve these aims our basic illustrative IBM could readily be extended in multiple ways to include: more zones and/or patches with different structures of seasonality and zone‐specific survival and/or reproductive success; different forms of density‐dependence and costs of migration and dispersal; different life histories, including iteroparity and age, sex, stage, state and/or cohort‐structure in any demographic rate; different forms of temporal (as well as spatial) variation in seasonality; different forms of plasticity, canalisation, social learning and assortative mating in migration *versus* residence; and different underlying genetic architectures. For example, the expression of migration *versus* residence could be explicitly modelled as a threshold trait, thereby formally linking quantitative genetic and population dynamic theory (e.g. Pulido, 2011; Cobben & Van Noordwijk, 2016).

## VI. REQUIREMENTS AND OPPORTUNITIES FOR EMPIRICAL DATA

In general, elucidating principles of population and evolutionary dynamics, and forecasting dynamics of specific systems, requires empirical estimates of key demographic rate means, variances and covariances to inform appropriate model structures and parameterisations (e.g. Coulson, Gaillard, & Festa‐Bianchet, 2005; Benton *et al.,* 2006; Sæther *et al.,* 2013; Small‐Lorenz *et al.,* 2013; Urban *et al.,* 2016; Gamelon *et al.,* 2017). The PMMP concept posits that population dynamics will depend on the forms of sex‐, age‐, stage‐, state‐ and location‐specific migration *versus* residence, on the degrees of developmental and environmental canalisation *versus* individual plasticity in migration, and on location‐specific relationships between the form of migration *versus* residence and survival, dispersal and reproduction (including complex carry‐over effects, e.g. Fig. 4). Evolutionary dynamics will further depend on additive genetic variances (and resulting heritabilities) in individual propensity for migration, and additive genetic covariances with survival, dispersal and reproduction. Key empirical objectives should consequently be to quantify such effects in diverse partially migratory systems (Table 2).

### (1) Current knowledge and requirements

It is well established that the degree and form of partial migration can vary among conspecific populations and change over time (e.g. Berthold, 1999; Pulido, 2007; Hebblewhite & Merrill, 2011; Mysterud *et al.,* 2011; Kerr & Secor, 2012; Singh *et al.,* 2012; Hodge *et al.,* 2014; Morita *et al.,* 2014; Boyle, 2017; Peters *et al.,* 2017). However, until recently, it has rarely been feasible to collect sufficient individual‐based data to quantify within‐individual and among‐individual variation in migration *versus* residence, and covariation with other demographic rates arising across different sexes, ages and life‐history stages. New tracking technologies and large‐scale mark–recapture endeavours and associated analytical methods are revolutionising such work, and recent cutting‐edge studies demonstrate that forms of demographic variation, covariation and structure that could create complex PMMP dynamics do occur in diverse species.

For example, partial migration can occur to similar degrees in both sexes [e.g. European shag *Phalacrocorax aristotelis* (Grist *et al.,* 2017); skylark *Alauda arvensis* (Hegemann, Marra, & Tieleman, 2015)] or be sex‐biased, with females [e.g. red‐spotted newt (Grayson *et al.,* 2011); tiger shark *Galeocerdo cuvier* (Papastamatiou *et al.,* 2013); blackbird (Fudickar *et al.,* 2013); Cory's shearwater (Catry *et al.,* 2013)], or occasionally males [e.g. wandering albatross, *Diomedea exulans* (Weimerskirch *et al.,* 2015); pochard, *Aythya farina* (Gourlay‐Larour *et al.,* 2014)], as the more migratory sex.

Partial migration can also vary with environmental conditions, population density and individual state [e.g. brown trout *Salmo trutta* (Olsson *et al.,* 2006); roach *Rutilus rutilus* (Brodersen *et al.,* 2008); red‐spotted newt (Grayson & Wilbur, 2009); elk *Cervus elaphus* (Hebblewhite & Merrill, 2011; Eggeman *et al.,* 2016); Cory's shearwater (Catry *et al.,* 2013); roe deer *Capreolus capreolus* (Peters *et al.,* 2017); Galapagos tortoises *Chelonoidis* sp. (Yackulic *et al.,* 2017)]. Extreme environmental events, such as intense rainfall or cold weather, can also trigger responsive partial migration [e.g. Newton, 2008; white‐ruffed manakin *Corapipo altera* (Boyle *et al.,* 2010); blackbird (Fudickar *et al.,* 2013)]. An individual's form of migration or residence can consequently be plastic, showing considerable within‐individual variation among years [e.g. red‐spotted newt (Grayson *et al.,* 2011); skylark (Hegemann *et al.,* 2015); elk (Eggeman *et al.,* 2016; see also Peters *et al.,* 2017)]. Conversely, migration and residence can be strongly canalised, defined as highly repeatable within individuals across different years and environmental conditions [e.g. European shag (Grist *et al.,* 2014); American dipper (Gillis *et al.,* 2008)]. Such high repeatability implies that substantial heritability, and underlying additive genetic variance, could potentially exist. Further, the degree of consistency in migration can also vary among individuals within populations [e.g. roach (Brodersen *et al.,* 2012)], implying that additive genetic variance in plasticity could also exist.

Diverse components of reproduction and/or survival can differ between migrants and residents, with contrasting effects observed in different species and systems. For example, resident American kestrels (*Falco sparverius*) bred earlier than migrants in two of three study years (Anderson *et al.,* 2015), and resident European shags bred earlier and more successfully than migrants in three successive years (Grist *et al.,* 2017). Resident American dippers also had higher breeding success than migrants, with no consistent difference in annual survival (Gillis *et al.,* 2008; Green *et al.,* 2015). Steelhead (*Oncorhynchus mykiss*) that undertook an additional early‐life return migration to freshwater were estimated to have lower fecundity than comparable individuals that had remained in the migrated oceanic state (Hodge *et al.,* 2014). Measures of reproductive success did not differ between resident and migrant skylarks, but return rates after subsequent winters were lower for residents (Hegemann *et al.,* 2015). Resident roach also had lower survival than migrants, due to higher predation rates (Skov *et al.,* 2013), and resident blackbirds had lower average survival probabilities than migrants across 7 years (Zúñiga *et al.,* 2017). By contrast, migrant elk had lower survival probabilities than residents, but had higher pregnancy rates and calf mass (Hebblewhite & Merrill, 2011). Survival was also higher in resident than migrant red‐spotted newts across a range of environmental conditions (Grayson *et al.,* 2011). Resident male newts also had higher reproductive success than migrants, while fecundity did not differ between resident and migrant breeding females but migrants were more likely to skip breeding (Grayson *et al.,* 2011).

Taken together, these diverse examples illustrate that the distinction between migration and residence can be associated with substantial demographic variation, which could be both causal and consequential (e.g. Fig. 4). However, as yet, no clear overarching or consistent patterns of demographic variation in relation to partial migration are evident across the available studies, and numerous key questions remain unanswered (Table 2). For example, few studies have quantified the ages or stages at which individuals from different sub‐populations develop or canalise different strategies of migration or residence, or hence quantified emerging age‐specificity or partitioned longitudinal (within‐individual) variation from cross‐sectional (among‐individual) variation stemming from selection (e.g. Singh *et al.,* 2012; Sergio *et al.,* 2014; Flack *et al.,* 2016). Indeed, few studies have quantified longitudinal variation in migration or residence in individual males and females across all key stage‐ or age‐classes, or hence explicitly quantified the degree of sex‐ or stage‐specific plasticity or canalisation of migration across varying environmental conditions (Eggeman *et al.,* 2016). Few studies have quantified relationships between migration, survival and reproduction across multiple years and sub‐populations, or thereby quantified the degree to which such relationships depend on spatio‐temporal variation in seasonality and environmental conditions (e.g. Hebblewhite & Merrill, 2011). No studies have yet directly quantified the lifelong fitness consequences of different strategies of migration *versus* residence (Gaillard, 2013). Relationships between dispersal and migration have been postulated but rarely quantified in free‐living partially migratory vertebrates (Berthold, 1999; Eggeman *et al.,* 2016), although natal dispersal was associated with winter habitat use in obligately migratory American redstarts (*Setophaga ruticilla*; Studds, Kyser, & Marra, 2008). Few studies have explicitly quantified carry‐over effects of extreme events on subsequent partial migration and associated demography, and such effects were not considered by recent reviews of biological responses to extreme climatic events (e.g. Van de Pol *et al.,* 2017). While the phenomena of responsive and irruptive migration are widely recognised (Berthold, 1999; Newton, 2008; Boyle *et al.,* 2010; Lindén *et al.,* 2011), the previous and subsequent life histories of individuals that do and do not irruptively migrate are typically unknown. In one interesting exception, male white‐ruffed manakins that migrated in response to intense rainfall subsequently achieved lower mating success than males that remained resident (Boyle *et al.,* 2011).

Regarding genetic variation, captive‐breeding experiments on birds have shown that the degree and timing of migratory activity (i.e. ‘nightrestlessness’) is highly heritable, and rapid switches in migration observed in wild populations further imply that substantial genetic variation exists (Berthold, 1999; Pulido *et al.,* 2001; Pulido, 2007; Pulido & Berthold, 2010; Liedvogel *et al.,* 2011). Breeding experiments also suggest that heritable migratory phenotypes have a polygenic basic and can consequently be considered as quantitative genetic threshold traits, where among‐individual variation in underlying ‘liability’ (i.e. latent propensity) for migration translates into discrete phenotypes (Pulido, 2007, 2011; Liedvogel & Lundberg, 2014). Similar experiments have estimated high heritability of the occurrence of seaward migration in Atlantic salmon (*Salmo salar*; Páez *et al.,* 2011), and of migration timing in chinook salmon (*Oncorhynchus tshawytscha*; Quinn *et al.,* 2000). Meanwhile, molecular genetic studies have detected associations between specific genotypes and migratory phenotypes, for example in admixed caribou (*Rangifer tarandus*) lineages (McDevitt *et al.,* 2009). However, there is also evidence for a substantial environmental basis to migration in birds and fish (e.g. Olsson *et al.,* 2006; Hodge *et al.,* 2014; Morita *et al.,* 2014), and among‐year individual repeatabilities can be low (e.g. Hegemann *et al.,* 2015). Overall, additive genetic variances and heritabilities in liability for migration, and genetic covariances and correlations with reproduction, survival and dispersal, have not yet been explicitly estimated in free‐living populations experiencing fully natural spatio‐temporal variation in environmental conditions, rather than in captive‐bred populations experiencing tightly controlled conditions with minimal environmental or socially induced variance (Pulido *et al.,* 2001; Van Noordwijk *et al.,* 2006; Pulido & Berthold, 2010; Liedvogel *et al.,* 2011; Páez *et al.,* 2011; Pulido, 2011).

### (2) Approaches to addressing empirical objectives

Progress in addressing general questions (Table 2), and in formulating and testing system‐specific hypotheses, requires different sets of migrants, residents and dispersers to be identified in systems where individual reproduction and survival, and environmental conditions and population density, can be quantified across single or multiple locations. This is most likely to be achieved by deploying appropriate tracking technologies and/or extensive observations within multi‐season and/or multi‐year field studies (e.g. Gillis *et al.,* 2008; Grayson *et al.,* 2011; Catry *et al.,* 2013; Skov *et al.,* 2013; Sergio *et al.,* 2014; Weimerskirch *et al.,* 2015; Eggeman *et al.,* 2016; Grist *et al.,* 2017; Zúñiga *et al.,* 2017, see also Marra *et al.,* 2015). Resulting data can be coupled with sophisticated statistical methods designed to minimise bias and quantify uncertainty in classifying diverse individual migration strategies and estimating associated demographic rates. These include analyses of remote‐tracking data (e.g. Papastamatiou *et al.,* 2013; Cagnacci *et al.,* 2016; Gurarie *et al.,* 2017), and multi‐state, multi‐event and integrated population models of mark–recapture or encounter data and associated population counts and observations of reproductive success (e.g. Gourlay‐Larour *et al.,* 2014; Cayuela *et al.,* 2017; Rushing *et al.,* 2017).

Beyond such observational studies, experiments that manipulate individual condition or local density can reveal reaction norms in migration including the form of density‐dependence (e.g. Brodersen *et al.,* 2008; Grayson & Wilbur, 2009). Such experiments could usefully be embedded in systems where individuals' subsequent life histories can be recorded. However, such experiments cannot directly quantify costs or benefits of migration, defined as ensuing changes in survival or reproduction, because these life‐history traits might be directly affected by the underlying manipulation. Indeed, there are few obvious ways in which the form or occurrence of migration can be directly manipulated independent of any other trait or state variable. In one tractable system, Rivrud *et al*. (2016) manipulated the spring release date (and hence migration time) of semi‐captive elk and showed that late‐released females accessed lower‐quality forage than early‐released females despite increased migration speed. However, any life‐history consequences of late release were not quantified. Such experiments should therefore be complemented by quantitative genetic analyses that utilise information on relatedness among observed individuals to separate genetic and environmental covariances underlying the occurrence of migration, and attribute environmental variances to individuals, families, years, cohorts and locations. Multiple recent developments in quantitative genetic analyses of wild population data will facilitate such decompositions of variation in liability for migration once sufficient data on individuals' seasonal locations can be collected. These include: methods for analysing non‐Gaussian traits, including threshold models for binary traits (Hadfield, 2010); accounting for genetic effects of immigration (Wolak & Reid, 2017); accounting for missing data resulting from selection and/or observation failure (Hadfield, 2008; Steinsland *et al.,* 2014), including by combining quantitative genetic and state‐space models (Papaïx *et al.,* 2010); and pedigree‐free (i.e. genomic) estimation (e.g. Bérénos *et al.,* 2014). In principle, such approaches could also estimate additive genetic variance in individual plasticity in migration, and estimate genetic covariances between migration and reproduction, survival and dispersal, and thereby directly quantify evolutionary drivers and constraints.

Overall, it may not be feasible, or relevant, to estimate all possible parameters and relationships pertaining to PMMP demography and dynamics (e.g. Fig. 4, Table 2) within single systems or studies. Such endeavours will certainly require greater spatial replication than is typically achieved in animal demography studies to date (e.g. Faaborg *et al.,* 2010; Singh & Leonardsson, 2014; Salguero‐Gómez *et al.,* 2016). However, once estimates accumulate across studies, meta‐analyses and comparative approaches might reveal general patterns of variation and covariation in and among migration *versus* residence and other demographic rates, as is increasingly being achieved for age‐ and sex‐specific variation in reproduction and survival (e.g. Coulson *et al.,* 2005; Sæther *et al.,* 2016; Salguero‐Gómez *et al.,* 2016), and for sex‐biased dispersal (e.g. Trochet *et al.,* 2016).

It has been emphasised that understanding and forecasting population dynamic responses to spatio‐temporal environmental change will ultimately require data‐informed models that link all key components of demographic variation to underlying environmental variation *via* key underlying mechanisms, and hence embrace all associated complexity (e.g. Thuiller *et al.,* 2013; Urban *et al.,* 2016; Cabral, Valente, & Hartig, 2017). In the context of partial migration, considerable recent work has focused on investigating proximate energetic, physiological, social, behavioural and ecological causes of individual migration *versus* residence, and thereby testing specific mechanistic hypotheses pertaining to individual size, condition, dominance and phenology (e.g. Olsson *et al.,* 2006; Brodersen *et al.,* 2008; Grayson & Wilbur, 2009; Jahn *et al.,* 2010; Chapman *et al.,* 2011, 2012; Harrison *et al.,* 2011; Hebblewhite & Merrill, 2011; Kokko, 2011; Páez *et al.,* 2011; Fudickar *et al.,* 2013; Hegemann *et al.,* 2015; Peters *et al.,* 2017; Yackulic *et al.,* 2017). Overarching future objectives should be to link such emerging mechanistic understanding with new knowledge of demographic variation, covariation, structure and heterogeneity involving partial migration, and thereby elucidate the population dynamic and ecosystem‐level consequences.

## VII. CONCLUSIONS

1. Key links between environmental and demographic variation, and resulting population and evolutionary dynamics, have not yet been elucidated in spatially structured populations where individuals vary in their expression of seasonal migration, even though such migration constitutes one pre‐eminent means by which individuals can respond to seasonal environmental variation and change.
2. Empirical studies are increasingly showing that many populations are partially migratory, where some individuals remain resident at one location all year round while other individuals make reversible seasonal migrations. Such partial migration could be structured by sex, age, stage, cohort and/or individual state or location, could covary with reproduction, survival and dispersal, and could show rapid evolutionary responses to seasonal selection.
3. However, existing population dynamic theory and models have not fully encompassed spatio‐temporal demographic variation involving partial migration. Rather, existing models consider demographic complexity in spatially structured populations with dispersal but no migration, or consider populations with full obligate migration, or consider basic scenarios of partial migration that are spatially and demographically simplistic.
4. We propose the concept of a ‘partially migratory meta‐population’ (PMMP) as a framework that can encompass all forms of spatio‐temporal demographic variation in partial migration alongside reproduction, survival and dispersal. We outline a series of hypotheses regarding forms of demographic covariation and structure, and eco‐evolutionary dynamics, that could arise in PMMPs.
5. General principles of PMMP dynamics could be elucidated through a hierarchy of modelling developments, thereby utilising tools that have previously been developed and applied to spatially structured populations with dispersal but no migration, or to populations with obligate migration. We present an evolutionary individual‐based model to illustrate one such approach, and show that multiple forms of partial migration can readily co‐exist even given a simple life history and spatial structure of seasonality.
6. New tracking technologies and extensive mark–recapture studies, and associated statistical capabilities, will increasingly facilitate empirical estimation of spatial and demographic structures of partial migration and associated demographic covariances and underlying genetic and environmental variances.
7. Together, resulting theoretical and empirical advances, alongside emerging understanding of physiological, behavioural and ecological mechanisms that shape the form of partial migration, should generate new holistic understanding of the implications of seasonal environmental variation and change, and evolving seasonal migration, for spatio‐temporal population dynamics. This understanding should provide a step change in our ability to forecast and effectively manage dynamics of numerous species that occupy spatially structured seasonally varying environments.

## VIII. ACKNOWLEDGMENTS

J. M. R. conceived the overarching ideas and wrote the manuscript; C. D. built and analysed the individual‐based model, with conceptual input from J. M. J. T. and J. M. R.; F. D., S. J. B., S. W. and J. M. J. T. contributed to idea development and manuscript editing. J. M. R., F. D., S. J. B. and J. M. J. T. acknowledge the support of NERC grant NE/R000859/1.

## Supporting information


**Appendix S1.** Details of an illustrative individual‐based model designed to examine population and evolutionary dynamics arising in partially migratory meta‐populations.
