# Supplementary material for: Population and evolutionary dynamics in spatially structured seasonally varying environments
Source: Biol Rev Camb Philos Soc. 2018 Mar 25;93(3):1578–603. doi: 10.1111/brv.12409 (PMC6849584; doi:10.1111/brv.12409)
Supplement: Supplementary file 1 — Appendix S1. Details of an illustrative individual‐based model designed to examine population and evolutionary dynamics arising in partially migratory meta‐populations. [file BRV-93-1578-s001.docx]

**Appendix S1. Details of an illustrative individual-based model designed to examine population and evolutionary dynamics arising in partially migratory meta-populations**

We modelled a world with three zones, which can respectively support reproduction but not non-breeding-season survival (zone A), non-breeding-season survival but not reproduction (zone C), or both seasonal activities (zone B, Fig. 5A). There are two identical patches within each zone (x and y) and hence six patches in total. However, due to the defined seasonality, only four patches can be functionally occupied in any one season.

We considered individuals that are haploid, asexual and semelparous. Each individual is born in a particular patch and then proceeds through four life-history phases: (*i*) move or not; (*ii*) survive the non-breeding season or not; (*iii*) move or not; and (*iv*) reproduction. All individuals then die. This set of four phases can generate all possible seasonal movement outcomes, comprising residence, dispersal and migration among patches and zones, and associated outcomes in terms of reproduction and survival. All individuals are identical except for their genetic movement propensities through phases *i* and *iii*, which are inherited from their parent.

Specifically, each individual carries eight genes, which together define the individual’s preferences and probabilities of between-zone and within-zone movements. To generate between-zone movements, each individual carries three genes that encode preferences to spend the non-breeding season (i.e. phase *ii*) in zone A, B or C, and three further genes that encode preferences to spend the breeding season (i.e. phase *iv*) in zone A, B or C. Each of the six genes can take numerical values between 0 and 1. During phases *i* and *iii*, each individual attempts to move to the zone for which it has the highest genetic preference value for the subsequent phase, or remains resident if it is already in its prefered zone. However, individuals that attempt to switch zones experience a survival cost *m*. Consequently, the probability of surviving a transition between spatially adjacent zones (i.e. A and B or B and C) is *p*_between_ = 1 – *m*. We assume that zone B lies between zones A and C and that mortality increases with distance travelled, so that individuals that attempt to move between zones A and C have a survival probability *p*_between_ = (1 – *m*)^2^. The inter-zone movement cost *m* was fixed within simulations but varied among simulations. For current illustrative purposes, we considered the range 0 ≤ *m* ≤ 0.25.

Each individual also carries two genes that directly encode the probability that it will switch between the x and y patches within zones (Fig. 5) during phases *i* and *iii*, respectively, thereby generating the possibility of within-zone dispersal. Each gene can take numerical values between 0 and 1, with within-zone movement realised as the outcome of a binomial trial. Individuals that attempt to switch patches experience a survival cost *t*, such that the probability of surviving a within-zone transition is *p*_within_ = 1 – *t*. For current purposes, we set *t* = 0.1 in all simulations.

Overall, an individual’s probability of surviving each transition phase *i* and *iii* is therefore *p*_Surv_ = *p*_between_ × *p*_within_, and equals 1 for individuals that do not move.

The numerical values of each individual’s eight movement-propensity genes are inherited from its (single) parent with a small probability of mutation. For current simulations, we set a mutation rate of 10^–4^ applied independently to each gene. When mutations occur, random mutation effect sizes for within-zone movement propensities were drawn from a uniform distribution [–0.05, 0.05] and added to the current value with propensity bounded at 0 and 1. For zone preferences, a random value was drawn from a uniform distribution spanning the full range [0, 1]. The values of the eight genes are independent, with no genetic linkage or pleiotropy.

Following phase *i*, an individual’s probability of non-breeding-season survival (i.e. phase *ii*) depends on which zone it occupies, and is density-dependent. Individuals in zone A die deterministically, while individuals in zones B and C survive with a probability that decreases linearly with increasing density as *p*_NB_ = 1 – *zN*, where *N* is the local population size in the focal individual’s current patch and *z* is a parameter that controls the strength of density-dependence. Individual survival or mortality was realised as the outcome of a binomial trial. The parameter *z* was fixed within simulations but varied among simulations. For current purposes, we considered the range 0.0001 ≤ *z* ≤ 0.0005, which translates to a 50% mortality rate for *N* = 1250 to 5000.

An individual’s reproductive success (i.e. phase *iv*) depends on which zone it occupies following phase *iii*, and is also density-dependent. Individuals in zone C deterministically fail to reproduce, while individuals in zones A and B produce a mean number of offspring per individual (*f*) defined by a Ricker model such that *f* = exp(*r* * (1 – *N* / *K*)), where *r* is the population growth rate, *N* is again the local population size in the focal individual’s current patch and *K* is a parameter that controls the strength of density-dependence. For each individual, realised reproductive success is drawn from a Poisson distribution with mean *f*. *K* and *r* are both variables but were set to 1000 and 2, respectively, for current illustrative simulations.

All simulations were initialised with a total population of 5000 individuals assigned randomly across patches. Each individual was allocated a random starting value for each of its eight movement-preference genes, with zone-preference values drawn from a uniform distribution [0, 1] and within-zone movement-propensity values drawn from a uniform distribution [0, 0.2].

Simulations were run for 20,000 generations. After the final generation, or a sequence of generations, the numbers of individuals that had enacted each of the four possible lifelong movement strategies (i.e. BBB, ABA, BCB and ACA), and the mean values of the two within-zone movement-propensity genes, were recorded.
